# Muscle Quality Responses of Pearl Gentian Grouper (*Epinephelus fuscoguttatus* ♀ × *Epinephelus lanceolatus* ♂) to Fishmeal Replacement by Defatted Silkworm Pupae Meal: Insights from Texture, Histology, Antioxidant Status and Metabolomics

**DOI:** 10.3390/foods15152640

**Published:** 2026-07-28

**Authors:** Yongkang Feng, Jian Chen, Qinglin Liu, Yudong Zheng, Zekai Xiao, Beiping Tan, Baogui Tang, Shuang Zhang

**Affiliations:** 1 College of Fisheries, Guangdong Ocean University, Zhanjiang 524088, China; 2 Zhanjiang Experimental Station, Chinese Academy of Tropical Agricultural Sciences, Zhanjiang 524013, China; 3 Aquatic Animals Precision Nutrition and High Efficiency Feed Engineering Research Center of Guangdong Province, Zhanjiang 524088, China

**Keywords:** defatted silkworm pupae meal, fishmeal substitution, pearl gentian grouper, flesh quality, metabolomics

## Abstract

This study investigated how defatted silkworm pupae meal (DSPM) modulates flesh quality in pearl gentian grouper (*Epinephelus fuscoguttatus* ♀ × *Epinephelus lanceolatus* ♂). A total of 360 size-uniform fish were randomly assigned to four dietary treatments for 8 weeks: D0, the fishmeal (FM) based control diet; and D1, D2, and D3, in which DSPM substituted 25%, 50%, and 100% of dietary FM, respectively, with three replicates per treatment and 30 fish per replicate. Compared with D0, D1 and D2 improved final body weight, weight gain rate, specific growth rate, and feed conversion ratio, whereas D3 reduced survival and feed utilization. For flesh quality, D2 showed the most favorable phenotype, characterized by higher crude protein, collagen, flavor-associated amino acids, pH, texture attributes, and muscle fiber density, together with lower ether extract, cooking loss, freezing loss, and shear force. DSPM substitution also increased unsaturated and polyunsaturated fatty acid proportions and improved lipid health indices. Moderate substitution, especially D2, enhanced oxidative stability by increasing SOD and CAT activities and reducing MDA accumulation. Non-targeted metabolomics combined with qPCR analysis of selected flesh quality-related genes suggested that these improvements were associated with osmotic regulation, collagen remodeling, purine metabolism, membrane lipid homeostasis, nutrient sensing, myogenesis, and antioxidant defense. Complete FM substitution impaired muscle structure, water retention, and redox balance. Thus, 50% FM substitution with DSPM effectively optimized grouper flesh quality in this study.

## 1. Introduction

Flesh quality is an important determinant of the nutritional, technological, and sensory value of farmed fish, especially for high-value aquaculture species whose market competitiveness increasingly depends not only on growth performance but also on edible quality. These quality attributes include muscle composition, structural and physicochemical properties, nutritional value, and sensory-related characteristics, which directly affect consumer acceptance, processing suitability, shelf-life stability, and economic value. Therefore, improving flesh quality has become an important objective in modern aquaculture nutrition, alongside the conventional goals of maximizing growth performance and feed efficiency [1].

These quality attributes are controlled by the coordinated changes in muscle protein deposition, collagen turnover, membrane lipid homeostasis, and metabolite accumulation, which are key areas of interest between aquaculture production and food chemistry [2]. Among the dietary factors affecting flesh quality, alternative protein sources, particularly insect-derived proteins, have received increasing attention because they provide amino acids for muscle accretion and may also influence connective tissue development, lipid deposition, oxidative stability, and flavor-related metabolite formation due to their high protein content, balanced nutrient composition, and potential functional compounds [3,4,5]. Accordingly, replacement of fishmeal (FM) with alternative protein sources has attracted increasing attention in aquaculture, not only because of the rising cost and limited availability of FM, but also because such replacement may substantially alter flesh quality traits, including proximate composition, amino acid and fatty acid profiles, texture, water-holding capacity, muscle histomorphology, and oxidative stability. However, their effects on flesh quality are often species-specific and strongly dependent on the type and inclusion level of insect meal; moderate replacement may improve nutrient deposition and flesh quality, whereas excessive replacement may impair digestibility, disturb nutrient balance, induce oxidative stress, or weaken tissue structure [6].

Defatted silkworm pupae meal (DSPM) is a promising protein component derived from the sericulture industry and contains protein fractions, essential amino acids, lipids, and bioactive substances that may influence muscle metabolism, nutrient deposition, and flesh quality formation [7]. Compared with some other insect protein sources, its defatted form may be more suitable for feed formulation because lipid reduction can improve storage stability and facilitate its use as a concentrated protein ingredient. Beyond its nutritional value, DSPM may regulate flesh quality by affecting protein deposition, collagen-related amino acid supply, lipid nutritional composition, antioxidant defense, and flavor-related biochemical pathways [8]. However, previous studies on insect proteins in aquatic animals have mainly focused on growth performance, feed utilization, digestion, and physiological responses, whereas their effects on flesh quality formation remain less understood.

Pearl gentian grouper (*Epinephelus fuscoguttatus* ♀ × *Epinephelus lanceolatus* ♂) is a high-value carnivorous marine fish whose flesh quality has considerable economic importance. At present, studies on FM replacement in grouper have mainly emphasized growth, feed utilization, intestinal physiology, or health status, whereas the regulatory mechanisms by which alternative protein sources affect flesh quality formation remain poorly characterized. In particular, evidence is still lacking on how DSPM influences muscle structural organization, collagen remodeling, flavor-related biochemical potential, lipid nutritional traits, and antioxidant homeostasis in this species [9,10]. These aspects are important because collagen content and muscle fiber characteristics are closely associated with texture and water-holding capacity, amino acids and purine-related metabolites contribute to flavor potential, fatty acid composition reflects lipid nutritional value and oxidative susceptibility, and antioxidant status is essential for maintaining muscle structure and delaying quality deterioration [11,12,13,14,15].

Therefore, this study evaluated the effects of graded replacement of FM by DSPM on growth performance and flesh quality in pearl gentian grouper. Fish were fed diets in which DSPM replaced 0%, 25%, 50%, or 100% of dietary FM. Muscle nutritional composition, physicochemical properties, histomorphology, antioxidant status, non-targeted metabolomics, and quality-related gene expression were analyzed to clarify the regulatory role of dietary DSPM in flesh quality formation. By integrating phenotypic, metabolic, and molecular evidence, this study aimed to provide a practical basis for the application of DSPM in grouper feeds and a broader reference for understanding how insect-derived proteins modulate flesh quality in carnivorous fish.

## 2. Materials and Methods

### 2.1. Ethical Statement

All experimental procedures were reviewed and approved by the College of Fisheries, Guangdong Ocean University (Approval No. GDOU-IACUC-2025-A0831). In accordance with these guidelines, all handling procedures for grouper were conducted so as to minimize animal suffering.

### 2.2. Experimental Diets and Design

Pearl gentian grouper used in this study were obtained from a local aquaculture farm in Zhanjiang, China. After a one-week acclimation period, 360 healthy fish with similar body weights were randomly selected and equally distributed into 12 glass tanks (1 m^3^). The fish were assigned to four dietary treatment groups, designated as D0, D1, D2, and D3. D0 served as the control group, while in the other three groups, DSPM (Jiangsu Duoyang Bioengineering Technology Co., Ltd., Changzhou, China) was used to replace 25%, 50%, and 100% of the FM in the basal diet, respectively. The analyzed proximate composition available for the DSPM batch used in this trial was as follows: moisture, 6.80%; crude protein, 69.95%; and crude fat, 4.70%. Each treatment consisted of three replicates, with 30 fish per replicate. The formulation of the basal diet is shown in Table S1.

The feeding trial was conducted at the Mazhang Base of the Zhanjiang Experimental Station, Chinese Academy of Tropical Agricultural Sciences, and lasted for 8 weeks. Fish were fed twice daily at 9:00 and 17:00. The initial feeding rate was set at 3–5% of body weight per day and was subsequently adjusted according to water temperature and feeding status. During the experiment, water quality was maintained within the following ranges: water temperature 22–28 °C, ammonia nitrogen < 0.05 mg/L, salinity 26–30 g/L, dissolved oxygen ≥ 6 mg/L, and pH 7.8–8.4. Two-thirds of the tank water was renewed daily to maintain stable water quality.

### 2.3. Growth Performance Analysis

At the end of the feeding trial, the fish were fasted for 24 h. The number of surviving fish in each tank was recorded, and their final body weights were measured. Based on the initial and final data, the following growth performance indices were calculated using the equations below:

(1) $$Survival\;Rates\;(SR,\%)\;=\;\frac{survival\;number}{initial\;number}\times 100$$

(2) $$Weight\;Gain\;Rate\;(WGR,\%)=\;\frac{\left(W2-W1\right)}{W1}\times 100$$

(3) $$Feed\;C\mathrm{onversion}\;R\mathrm{atios}\;(FCR)\;=\;\frac{F}{\left(W2-W1\right)}$$

(4) $$Specific\;Growth\;R\mathrm{ate}\;(SGR,\%\;d^{-1})\;=\;\frac{\left(\ln W2-\ln W1\right)}{T}\times 100$$

In the above equations: *W*1 and *W*2 represent the initial body weight (IBW, g) and final body weight (FBW, g), respectively, *T* represents the experimental duration (days), and *F* represents the feed intake of fish during the experimental period (g).

### 2.4. Sample Collection

At the end of the feeding trial, 21 fish were randomly sampled from each tank and anesthetized with 0.1 g/L of MS-222 (Sigma, St. Louis, MO, USA) solution. Dorsal muscle tissues from six fish were first collected immediately after dissection and used for the determination of texture properties, cooking loss, and shear force. Dorsal muscle samples from another three fish were rapidly transferred into EP tubes containing RNA-later (Invitrogen, Thermo Fisher Scientific, Waltham, MA, USA) and stored at −80 °C for the analysis of related gene expression. Subsequently, muscle samples from four fish were collected into 1 mL cryotubes and stored at −80 °C for enzyme activity assays. In addition, muscle samples from another six fish were collected and stored at −80 °C for non-targeted metabolomic analysis. Finally, dorsal muscle tissues from two fish were fixed in 10 mL EP tubes containing 4% paraformaldehyde (Seville Biotechnology Co., Ltd., Wuhan, China) for histomorphological analysis. Dorsal muscle from the remaining fish was collected and temporarily stored at −80 °C for subsequent analyses of proximate composition, freezing loss, pH, amino acid composition, and fatty acid profiles.

### 2.5. Determination of Muscle Nutritional Components

Muscle samples were collected from the dorsal muscle, homogenized, and used for the determination of proximate composition, hydroxyproline content, amino acid composition, and fatty acid profiles. Moisture content was determined by oven drying at 105 °C to constant weight, crude protein by the Kjeldahl method, ether extract by Soxhlet extraction, and ash by incineration in a muffle furnace (Thermo Scientific, Waltham, MA, USA) at 550 °C. Hydroxyproline content was determined using a commercial assay kit (A030-2-1, Nanjing Jiancheng Bioengineering Institute, Nanjing, China). Muscle samples were homogenized and analyzed according to the manufacturer’s instructions, and collagen content was calculated by multiplying the hydroxyproline content by 8 [16]. Amino acid composition was determined according to GB 5009.124-2016 [17] using a HITACHI L-8900 high-speed amino acid analyzer (Hitachi, Tokyo, Japan), and fatty acid composition was determined according to GB 5009.168-2016 [18] using an Agilent 7890A gas chromatograph (Agilent Technologies, Santa Clara, CA, USA). The lipid health-related indices were calculated based on the fatty acid composition. The lipid health-related indices were calculated as follows:

(5) $$AI=\frac{4\times C14:0+C16:0}{\sum UFA}$$

(6) $$TI=\frac{C14:0+C16:0+C18:0}{0.5\sum MUFA+0.5\sum n\text{-}6PUFA+3\sum n\text{-}3PUFA+\frac{\sum n\text{-}3PUFA}{\sum n\text{-}6PUFA}}$$

### 2.6. Analysis of Muscle Physicochemical Properties

Muscle WHC was determined according to the method described by Zheng et al. [19]. One portion of each muscle sample was subjected separately to cooking treatment and freezing treatment. For the cooking-treated samples, fish muscle (H1) was wrapped in coarse cotton cloth and heated in a constant-temperature water bath at 90 °C for 5 min. The samples were then cooled to room temperature, surface moisture was removed by blotting, and the samples were weighed (H2). After cooling, the same samples were used for shear force determination. For the freezing-treated samples, fish muscle (*H*_1_) was frozen at −80 °C for 24 h, thawed at room temperature, blotted dry, and weighed (*H*_2_). Water loss was calculated as follows:

(7) $$Water\;Loss\;Rate\;(\%)\;=\frac{\left(H_{1}-H_{2}\right)}{H_{1}}\times 100$$

For pH determination, dorsal muscle samples were frozen at −80 °C for 24 h and then thawed to room temperature before measurement. Muscle pH was measured using a calibrated pH probe (Testo 205 pH meter, Testo AG, Lenzkirch, Germany). Muscle texture properties, including hardness, adhesiveness, springiness, chewiness, gumminess, cohesiveness, and resilience, as well as shear force, were measured using a TA.XTC-18 texture analyzer (Bosin Tech, Shanghai, China). For shear force measurement, each sample was sheared three times, with the cutting direction perpendicular to the orientation of the muscle fibers, and the peak shear force was used for statistical analysis. The texture analysis parameters were set as follows: cylindrical probe diameter, 25 mm, trigger force, 5 gf, test speed, 1 mm/s, and compression deformation, 60%. The program was set to hold the pressure for 2 s, with a 5 s interval between consecutive measurements.

### 2.7. Analysis of Muscle Histomorphology

After fixation for 24 h, muscle tissues were trimmed into blocks of approximately 10 × 5 × 5 mm^3^ and processed into paraffin sections, stained with hematoxylin and eosin (H&E), and sealed with neutral balsam. The sections were observed and imaged under an optical microscope (Nikon Corporation, Tokyo, Japan). After image acquisition, the number, density, and diameter of muscle fibers were quantified using ImageJ software (version 1.54).

### 2.8. Analysis of Muscle Antioxidant Enzyme Activities

The muscle tissue was homogenized on ice and centrifuged to obtain the supernatant, which was used to analyze the following indicators: Total antioxidant capacity (T-AOC, A015-2-1), superoxide dismutase (SOD, A001-3-2), catalase (CAT, A007-1-1), and malondialdehyde (MDA, A003-1-2), which were determined using commercial assay kits provided by Nanjing Jiancheng Bioengineering Institute (Nanjing, China). All procedures were performed strictly in accordance with the manufacturer’s instructions.

### 2.9. Muscle Non-Targeted Metabolomic Analysis

After thawing at 4 °C, the muscle samples were subjected to metabolomic analysis. An appropriate amount of each sample was added to a precooled methanol/acetonitrile/water solution (2:2:1, *v*/*v*), vortex-mixed, ultrasonicated at low temperature for 30 min, and then allowed to stand at −20 °C for 10 min. The mixture was centrifuged at 14,000× *g* for 20 min at 4 °C, and the supernatant was collected. The supernatant was dried under vacuum and reconstituted in 100 μL acetonitrile/water (1:1, *v*/*v*), vortex-mixed, and centrifuged again at 14,000× *g* for 15 min at 4 °C. A 2 μL aliquot of each sample was separated using a Vanquish UHPLC system (Thermo Fisher Scientific, Waltham, MA, USA) equipped with a HILIC column, and both MS and MS/MS data were acquired using an Orbitrap Exploris™ 480 mass spectrometer (Thermo Fisher Scientific, USA). Raw data were converted into MzML format using ProteoWizard software (version 3.0), followed by peak alignment, retention time correction, and peak area extraction using XCMS. Data acquired in positive (POS) and negative (NEG) ion modes were analyzed separately and then combined. All data were validated through quality control (QC) and quality assurance (QA) procedures. Principal component analysis (PCA) and partial least squares discriminant analysis (PLS-DA) were performed using the ropls package (version 1.44.0) in R (version 4.6.0). Differential metabolites between groups were identified based on the variable importance in projection (VIP) values from PLS-DA and *p* values from the *t*-test. Metabolites with VIP ≥ 1 and *p* < 0.05 were considered significantly different. These metabolites were further analyzed by fold change and visualized using volcano plots. The abundance of differential metabolites in each group was standardized by z-score normalization and visualized according to VIP values. Pathway enrichment analysis was performed based on the Kyoto Encyclopedia of Genes and Genomes (KEGG) database.

### 2.10. Gene Expression Analysis

Total RNA was isolated from grouper muscle using TRIzol reagent (Beijing QuanShiJin Biotechnology Co., Ltd., Beijing, China). RNA concentration was measured using a Thermo NanoDrop 2000 spectrophotometer (Thermo Fisher Scientific, USA). Total RNA was reverse transcribed into cDNA using the Evo M-MLV RT Kit with gDNA Clean for qPCR II (Accurate Biotechnology (Hunan) Co., Ltd., Changsha, China). Primer sequences were designed based on NCBI database information and are listed in Table S2. All primers were synthesized by Sangon Biotech Co., Ltd. (Shanghai, China). Quantitative real-time PCR (qPCR) was performed using the SYBR^®^ Green Premix Pro Taq HS qPCR Kit II (Accurate Biotechnology (Hunan) Co., Ltd., China) on a LightCycler 480 system (Roche Diagnostics, Rotkreuz, Switzerland) to determine the expression levels of *AMPD1*, *NT5E*, *AK1*, *CHDH*, *SLC7A5*, *MTOR*, *COL1α1*, *COL1α2*, *LOX*, *MyoD1*, *MyoG*, *GDF-8*, *FAS*, *SCD*, *FABP3*, *AMPK*, *CAPN1*, *CAST*, *SOD*, *GPX1α*, *CAT*, *Nrf2*, *HSP70*, *HSP90*, *PKMA*, and *LDHA*. The qPCR reactions were carried out following a standard two-step protocol, consisting of an initial denaturation at 95 °C for 30 s, followed by 40 cycles of 95 °C for 10 s and 60 °C for 30 s for combined annealing and extension. *β-actin* was used as the reference gene, and relative gene expression levels were calculated using the 2^−ΔΔCt^ method.

### 2.11. Statistical Analysis

Data are presented as the mean ± standard deviation (SD). Statistical differences among groups were evaluated by one-way analysis of variance (ANOVA) using SPSS Statistics 26.0 software (IBM Corp., Armonk, NY, USA). When a significant difference was detected (*p* < 0.05), Duncan’s multiple range test was performed for post hoc comparisons. In addition, Pearson’s correlation analysis was conducted to assess associations among variables, and the false discovery rate (FDR) correction was applied to identify significantly correlated indicators.

## 3. Results

### 3.1. Growth Performance

Dietary replacement of FM with DSPM significantly affected the growth performance of grouper, as shown in Table 1. Compared with the D0 group, SR in the D3 group was significantly decreased (*p* < 0.05), whereas no significant differences were observed in the D1 or D2 groups (*p* > 0.05). The FBW, WGR and SGR in the D1 and D2 groups were significantly higher than those in the control group D0 (*p* < 0.05), while no significant differences in FBW, WGR, or SGR were found between the D3 and D0 groups (*p* > 0.05). The FCR in the D1 and D2 groups was significantly lower than that in the D0 group (*p* < 0.05), whereas the FCR in the D3 group was significantly higher than that in the D0 group (*p* < 0.05).

### 3.2. Muscle Proximate Composition

As shown in Table 2, dietary replacement of FM with DSPM affected the proximate composition of grouper muscle. Compared with D0, moisture content was significantly higher in D2 and D3, whereas D1 did not differ significantly from D0. Crude protein content was significantly increased in all DSPM-fed groups (*p* < 0.05). Ether extract content was significantly decreased in D2 and D3, whereas no significant difference was observed between D1 and D0. Crude ash content was significantly elevated in D2 and D3 (*p* < 0.05). Notably, collagen content was significantly increased in D1 and D2, with the highest level observed in D2, while no significant difference was detected between D3 and D0 (*p* > 0.05).

### 3.3. Muscle Amino Acid Composition

**Figure 1:**
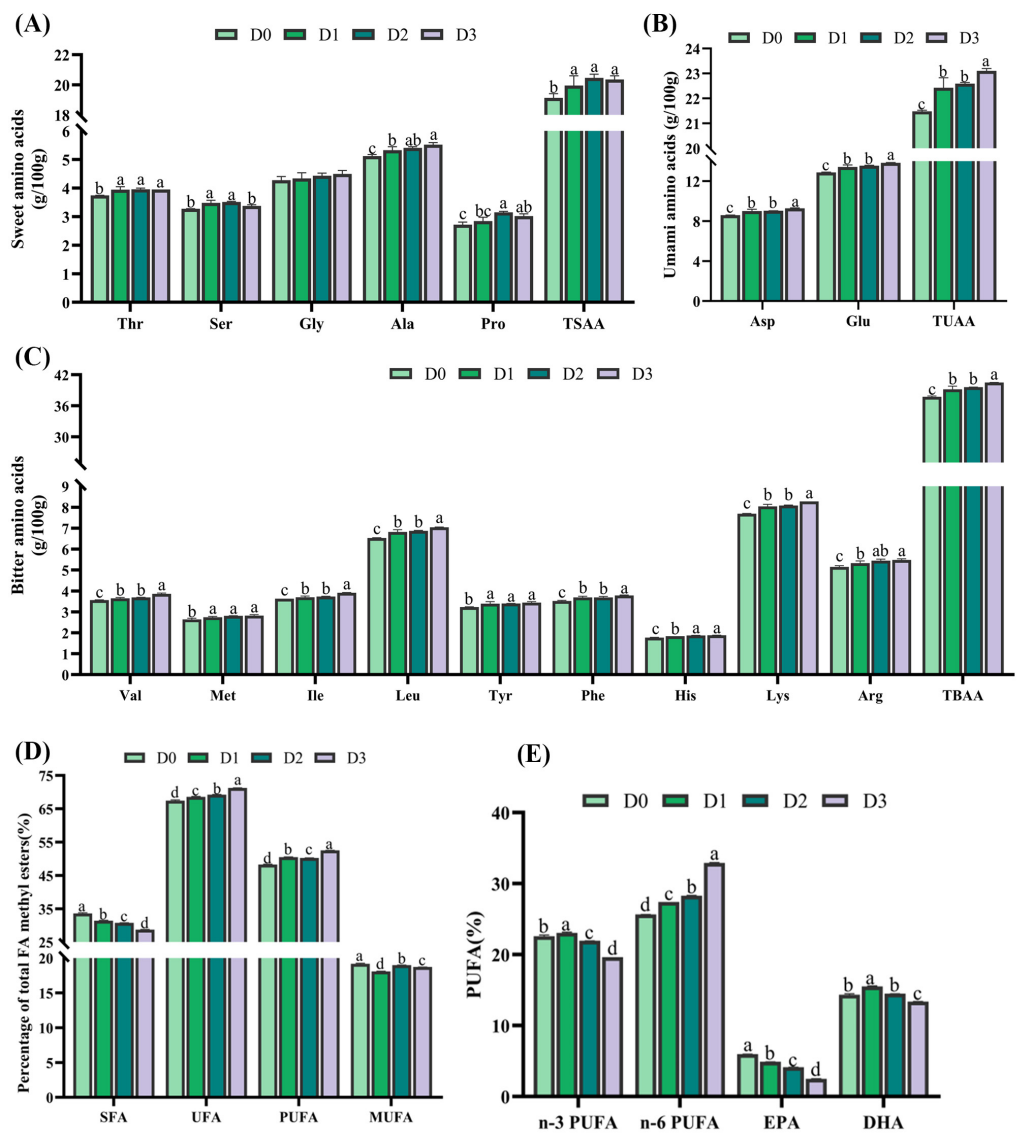
The muscle amino acid and fatty acid composition of pearl gentian grouper (*Epinephelus fuscoguttatus* ♀ × *Epinephelus lanceolatus* ♂). (**A**) Sweet amino acid profile. (**B**) Umami amino acid profile. (**C**) Bitter amino acid profile. (**D**) The fatty acid (FA) profile. (**E**) The polyunsaturated fatty acid (PUFA) profile. Notes: Data were presented as the mean ± SD, n = 3. Superscripts with different lowercase letters indicate significant differences between groups (*p* < 0.05).

Dietary DSPM replacement significantly enhanced amino acid deposition in grouper muscle, particularly with respect to flavor-related amino acids. As shown in Figure 1A–C, the contents of total sweet, umami, and bitter amino acids were all significantly increased in the DSPM replacement groups compared with D0 (*p* < 0.05). Among sweet amino acids, threonine (Thr), alanine (Ala), and proline (Pro) were consistently elevated in the DSPM replacement groups, while serine (Ser) was significantly increased in D1 and D2. Among umami amino acids, both aspartic acid (Asp) and glutamic acid (Glu) were significantly higher in all DSPM replacement groups than in D0, with the highest levels generally observed under the higher replacement levels. Among bitter amino acids, valine (Val), methionine (Met), isoleucine (Ile), leucine (Leu), lysine (Lys), and arginine (Arg) were significantly increased in the DSPM replacement groups, especially in D3. Consistently, the contents of total amino acids (TAA), total essential amino acids (TEAA), and total non-essential amino acids (TNEAA) were all significantly higher in the DSPM replacement groups than in the control group (*p* < 0.05, Table S3).

### 3.4. Muscle Fatty Acid Composition

Dietary DSPM replacement significantly modified the muscle fatty acid composition and overall improved lipid nutritional characteristics. As shown in Figure 1D, the DSPM replacement groups had significantly lower levels of total saturated fatty acids (ΣSFA) and total monounsaturated fatty acids (ΣMUFA), together with significantly higher levels of total unsaturated fatty acids (ΣUFA) and total polyunsaturated fatty acids (ΣPUFA), than D0 (*p* < 0.05). For key nutritional fatty acids, significant differences were observed in n-3 polyunsaturated fatty acids (n-3 PUFA), n-6 polyunsaturated fatty acids (n-6 PUFA), eicosapentaenoic acid (EPA), and docosahexaenoic acid (DHA) among groups (*p* < 0.05, Figure 1E). The D1 group showed the highest n-3 PUFA and DHA contents, whereas n-6 PUFA increased and EPA decreased with increasing DSPM replacement. Moreover, all DSPM replacement groups showed significantly lower atherogenic index (AI) and thrombogenic index (TI) values than the control group (*p* < 0.05, Table S4).

### 3.5. Muscle Physicochemical Properties

**Figure 2:**
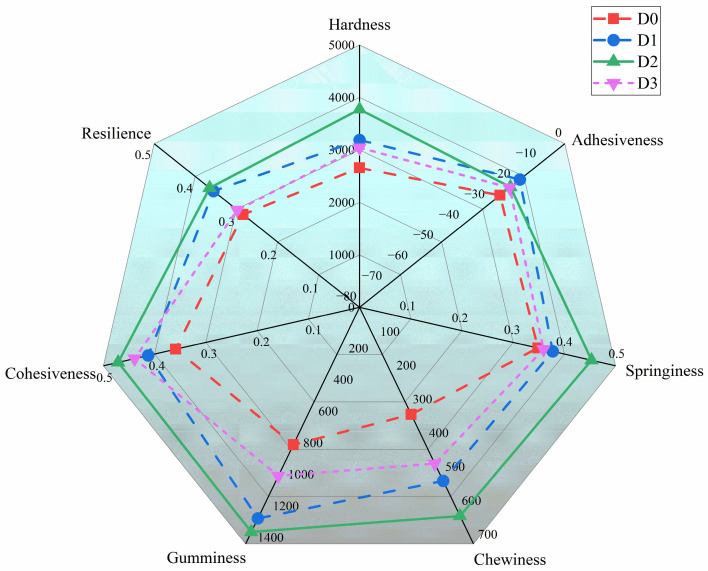
Radar chart of muscle texture. Notes: The radar chart shows the overall changes in hardness, adhesiveness, springiness, chewiness, gumminess, cohesiveness and resilience among the groups.

Dietary replacement of FM with DSPM improved the physicochemical properties of grouper muscle, with D2 showing the most favorable overall performance. As shown in Table 3, cooking loss and shear force were significantly reduced in all DSPM treatment groups compared with D0 (*p* < 0.05). Freezing loss was significantly lower in D2 than in D0, whereas D1 and D3 did not differ significantly from D0; however, D3 showed significantly higher freezing loss than D1 and D2. Thawed muscle pH was significantly higher in D1 and D2 than in D0, with the highest value observed in D2, whereas no significant difference was observed between D3 and D0. Texture profile analysis showed that most texture parameters were improved by DSPM inclusion, and D2 exhibited the highest values for hardness, springiness, chewiness, gumminess, cohesiveness, and resilience (Figure 2).

### 3.6. Muscle Histological Characteristics

**Figure 3:**
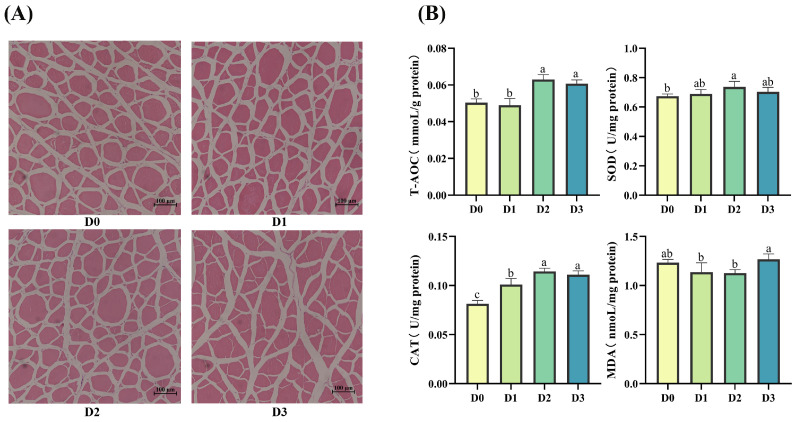
The muscle histomorphology and antioxidant status of pearl gentian grouper (*Epinephelus fuscoguttatus* ♀ × *Epinephelus lanceolatus* ♂). (**A**) HE-stained sections of fish dorsal muscle. (**B**) Muscle antioxidant-related indices. Notes: Data were presented as the mean ± SD, n = 3. Superscripts with different lowercase letters indicate significant differences between groups (*p* < 0.05).

Histological observation showed that moderate DSPM replacement improved muscle microstructure, whereas excessive replacement impaired it. Compared with D0, the D1 and D2 groups exhibited clearer fiber boundaries, a more compact arrangement, narrower endomysial spaces, and a more intact fascicular structure, whereas D3 showed looser fiber organization and widened connective tissue spaces (Figure 3A). Quantitative analysis further confirmed these structural changes. Muscle fiber density was significantly increased in D1 compared with D0, whereas D2 and D3 did not differ significantly from D0; however, D3 showed significantly lower muscle fiber density than D1 and D2. Muscle fiber diameter was significantly decreased in D1 and increased in D3 compared with D0, while no significant difference was observed between D2 and D0 (*p* < 0.05, Table 4). No significant difference in muscle fiber number was detected among groups (*p* > 0.05).

### 3.7. Muscle Antioxidant Enzyme Activities

Dietary DSPM replacement enhanced the antioxidant status of grouper muscle, although the response differed with replacement level. As shown in Figure 3B, SOD and CAT activities were significantly increased in all DSPM treatment groups compared with D0 (*p* < 0.05), and D2 generally showed the strongest antioxidant response. T-AOC was significantly elevated in D2 and D3, whereas MDA content was significantly reduced in D1 and D2 but markedly increased in D3 (*p* < 0.05), indicating that moderate DSPM inclusion improved redox balance, while excessive replacement promoted oxidative stress.

### 3.8. Non-Targeted Metabolomics Analysis of Muscle

Based on growth and flesh quality performance, the D0, D2, and D3 groups were selected for metabolomic analysis, with six muscle samples collected from each group. D0, D2, and D3 represented the basal, optimal, and excessive FM replacement states, respectively. D1 was excluded because its responses were intermediate and generally similar to those of D2. Thus, these three groups were sufficient to capture the major metabolic changes induced by graded DSPM replacement.

#### 3.8.1. Comparison and Analysis of Samples Among Groups

After low-quality peaks were filtered out, 5060 mass spectral features were obtained in the positive ion mode and 6549 in the negative ion mode. A total of 1834 annotated metabolites were identified based on the MS and MS/MS databases, including 894 metabolites in the positive ion mode and 940 in the negative ion mode (Table S5).

#### 3.8.2. Differential Metabolite Analysis

**Figure 4:**
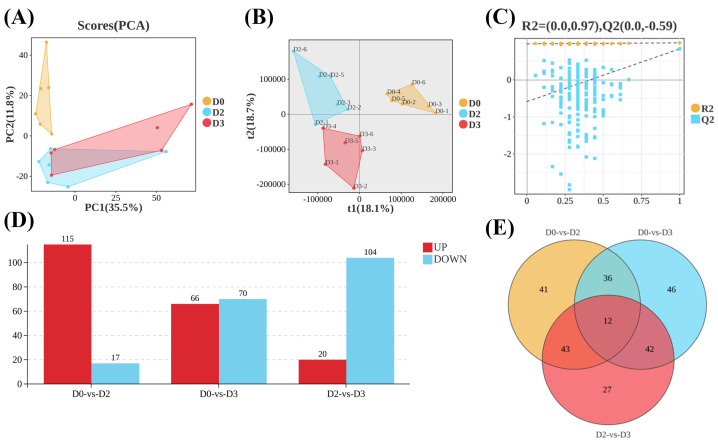
Multivariate statistical analysis and inter-group comparison of differential metabolites. (**A**) PCA score plot of metabolites from different groups. (**B**) PLS-DA score plot of metabolites from different groups. (**C**) PLS-DA permutation test plot. (**D**) Number of differential metabolites identified among pairwise comparisons. (**E**) Venn diagram of differential metabolites among groups.

As shown in Figure 4A,B, the PCA and PLS-DA results showed good sample clustering and clear metabolic differentiation among groups. The permutation test yielded an R^2^ value of 0.97 and a Q^2^ intercept of approximately −0.59, indicating good model fitness and predictive reliability with a low risk of overfitting (Figure 4C). As shown in Figure 4D, 132 differential metabolites were identified in D0 vs. D2, of which 17 were downregulated and 115 were upregulated. In D0 vs. D3, 136 differential metabolites were identified, including 70 downregulated and 66 upregulated metabolites. In D2 vs. D3, 124 differential metabolites were identified, including 104 downregulated and 20 upregulated metabolites. As shown in Figure 4E, the Venn diagram revealed partial overlap among the three comparisons, with 12 common differential metabolites. The numbers of comparison-specific differential metabolites were 41 for D0 vs. D2, 46 for D0 vs. D3, and 27 for D2 vs. D3.

**Figure 5:**
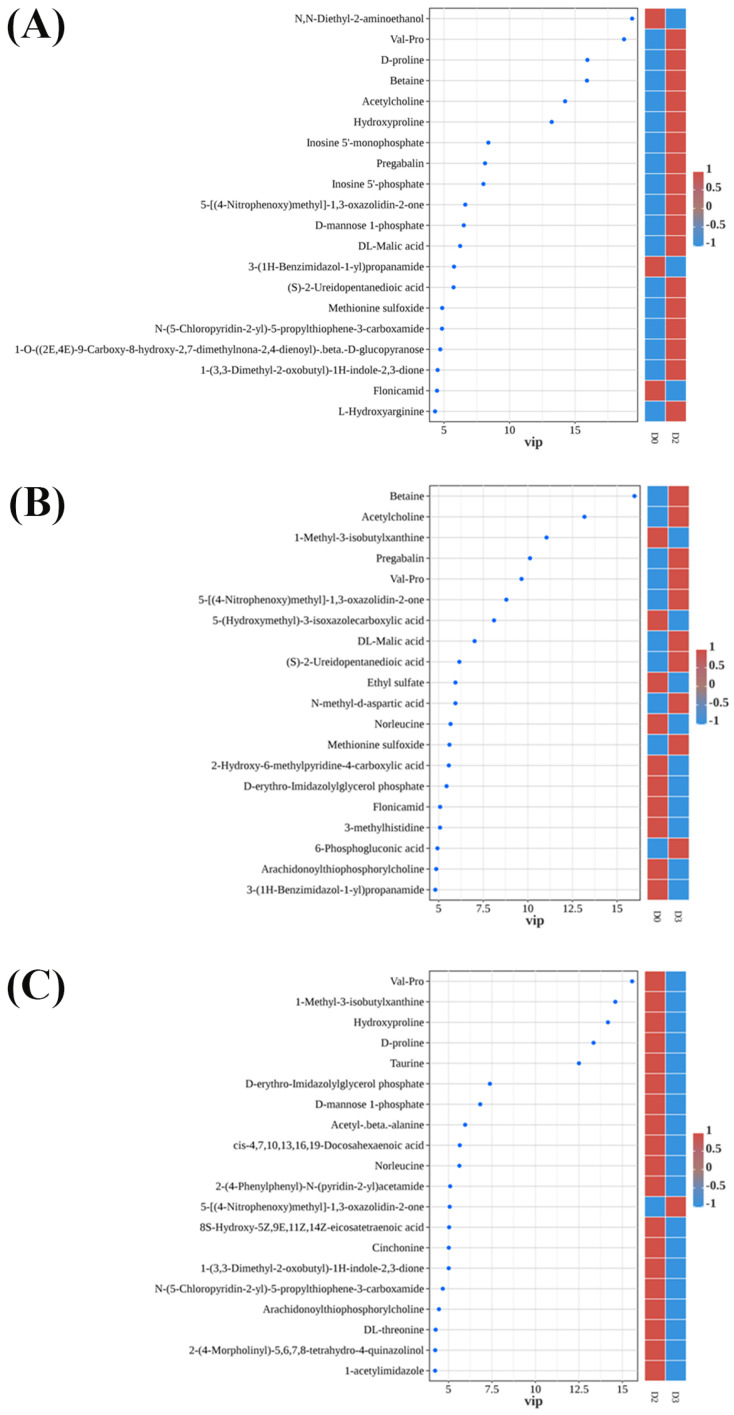
Identification and visualization of differential metabolites the top 20 ranked by VIP scores in pairwise comparisons. (**A**) Heatmap of differential metabolites in D0 vs. D2. (**B**) Heatmap of differential metabolites in D0 vs. D3. (**C**) Heatmap of differential metabolites in D2 vs. D3.

Based on the combined evaluation of VIP values from the PLS-DA model and volcano plots, the contribution and variation patterns of differential metabolites differed markedly among comparisons (*p* < 0.05). The top 20 metabolites ranked by VIP in D0 vs. D2 are shown in Figure 5A, mainly including N,N-Diethyl-2-aminoethanol, Val-Pro, D-proline, betaine, acetylcholine, hydroxyproline, and inosine 5′-monophosphate, indicating that amino acids/peptides, methyl donor-related metabolites, and nucleotide-related metabolites contributed strongly to group discrimination. The top 20 VIP-ranked metabolites in D0 vs. D3 are shown in Figure 5B, including betaine, acetylcholine, 1-methyl-3-isobutylxanthine, pregabalin, Val-Pro, DL-malic acid, methionine sulfoxide, 3-methylhistidine, and 6-phosphogluconic acid. Among these, metabolites related to energy/carbohydrate metabolism intermediates, oxidative modification of amino acids, and muscle protein degradation made major contributions to group discrimination. In D2 vs. D3, the top 20 metabolites ranked by VIP are shown in Figure 5C and mainly included Val-Pro, 1-methyl-3-isobutylxanthine, hydroxyproline, D-proline, taurine, and D-mannose 1-phosphate, suggesting that peptide/amino acid metabolism, osmoregulation-related metabolites, and fatty acid-related metabolites contributed greatly to the separation between D2 and D3.

Based on these results, a more stringent screening threshold of VIP > 5 and |log_2_FC| > 1 was further applied to identify representative differential metabolites (Table 5). Under this criterion, 7 differential metabolites were identified in D0 vs. D2 (6 upregulated and 1 downregulated), 8 in D0 vs. D3 (3 upregulated and 5 downregulated), and 6 in D2 vs. D3 (all downregulated). These results were consistent with the overall trends observed in the volcano plots, indicating that the differential metabolites were not only representative in terms of contribution, but also exhibited comparison-specific directional changes among treatment groups.

#### 3.8.3. KEGG Pathway Analysis

**Figure 6:**
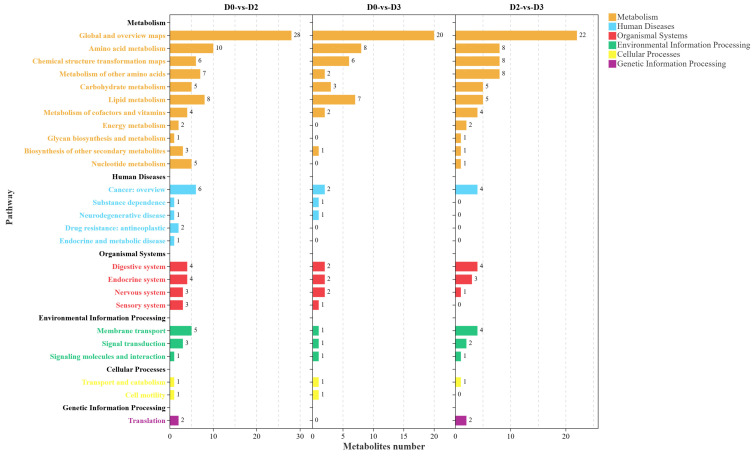
KEGG pathway enrichment analysis of differential metabolites. Notes: The vertical axis represents enriched metabolic pathways, and the horizontal axis represents the number of metabolites in each pathway.

KEGG enrichment analysis showed that the differential metabolites were mainly involved in pathways related to amino acid utilization, membrane lipid remodeling, and redox-associated carbon metabolism, rather than being broadly distributed across general functional categories. Although no canonical collagen-specific pathway was directly enriched, the enrichment of amino acid biosynthesis, protein digestion and absorption, and aminoacyl-tRNA biosynthesis, together with the differential abundance of hydroxyproline-related metabolites, suggested that dietary DSPM replacement affected collagen turnover and structural protein deposition in muscle (Figure 6).

**Figure 7:**
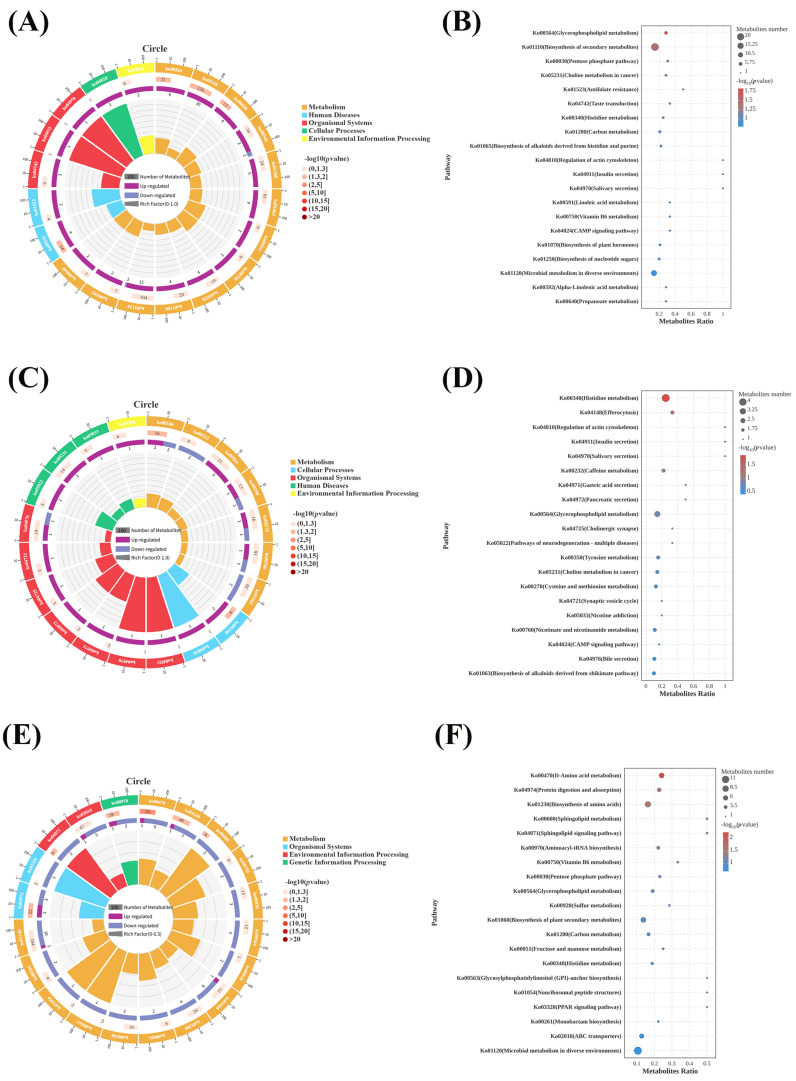
Enrichment analysis of metabolic pathways associated with differential metabolites in pairwise comparisons. (**A**) Circular plot of pathway enrichment analysis for D0 vs. D2. (**B**) Bubble plot of significantly enriched pathways for D0 vs. D2. (**C**) Circular plot of pathway enrichment analysis for D0 vs. D3. (**D**) Bubble plot of significantly enriched pathways for D0 vs. D3. (**E**) Circular plot of pathway enrichment analysis for D2 vs. D3. (**F**) Bubble plot of significantly enriched pathways for D2 vs. D3.

In D0 vs. D2, the enriched pathways were mainly associated with membrane lipid remodeling and redox-related metabolism, including glycerophospholipid metabolism, linoleic acid metabolism, alpha-linolenic acid metabolism, the pentose phosphate pathway, carbon metabolism, histidine metabolism, and vitamin B6 metabolism (Figure 7A,B). These results indicate that moderate DSPM replacement promoted the metabolic basis for membrane stability, lipid nutritional quality, and antioxidant regulation, while also enhancing amino acid-related metabolism associated with flesh quality formation.

In D0 vs. D3, the enriched pathways were mainly concentrated in amino acid metabolism and stress-related metabolic responses, including histidine metabolism, tyrosine metabolism, cysteine and methionine metabolism, and glycerophospholipid metabolism, together with several secretion- and neuro-related pathways (Figure 7C,D). Among these, the involvement of cysteine and methionine metabolism is particularly relevant to redox regulation, whereas the enrichment of glycerophospholipid metabolism suggests altered membrane lipid homeostasis under excessive DSPM replacement. These changes imply that complete FM replacement induced a less stable metabolic state that may be unfavorable for maintaining muscle quality.

In D2 vs. D3, the enriched pathways were mainly related to amino acid utilization/protein turnover, membrane lipid metabolism, and carbohydrate-associated redox metabolism, including D-amino acid metabolism, biosynthesis of amino acids, aminoacyl-tRNA biosynthesis, protein digestion and absorption, sphingolipid metabolism, the sphingolipid signaling pathway, glycerophospholipid metabolism, the PPAR signaling pathway, the pentose phosphate pathway, and fructose and mannose metabolism (Figure 7E,F). These pathways collectively suggest that, compared with D3, the D2 group had a greater capacity for amino acid incorporation and structural protein deposition, more coordinated membrane lipid remodeling, and better metabolic support for oxidative balance.

Overall, these KEGG results suggest that the metabolic differences among groups were mainly associated with collagen-related amino acid utilization, membrane lipid remodeling, and redox-associated metabolic regulation.

#### 3.8.4. Screening of Metabolomics-Associated Genes Related to Flesh Quality

**Figure 8:**
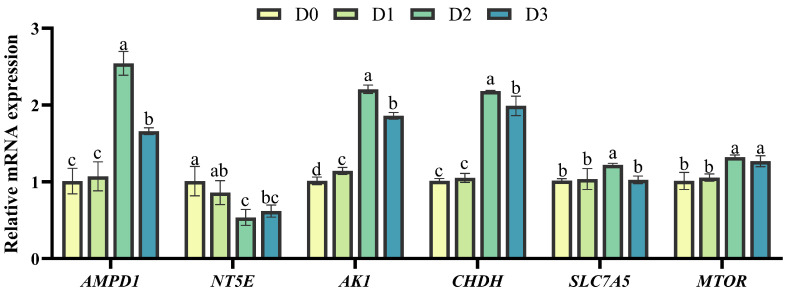
Relative mRNA expression levels of genes related to energy metabolism, amino acid transport and MTOR signaling in different groups of muscles. Notes: Data were presented as the mean ± SD, n = 3. Superscripts with different lowercase letters indicate significant differences between groups (*p* < 0.05).

Based on the metabolomic results, genes related to purine/energy metabolism and the MTOR signaling pathway were further analyzed in muscle tissue. As shown in Figure 8, moderate DSPM replacement, particularly in the D2 group, was characterized by the most active overall transcriptional response of genes involved in nucleotide turnover, methyl donor metabolism, amino acid transport, and nutrient sensing. Specifically, the expression levels of *AMPD1*, *AK1*, *CHDH*, and *SLC7A5* were all significantly upregulated in D2, with D2 showing the highest levels among groups (*p* < 0.05). Among these, *AMPD1* and *CHDH* were also significantly higher in D3 than in D0 and D1 (*p* < 0.05), whereas *AK1* displayed a stepwise increase from D0 to D2, with all intergroup differences being significant (*p* < 0.05). In contrast, *NT5E* showed an opposite trend, with the highest expression in D0 and the lowest in D2 (*p* < 0.05), while D1 and D3 remained at intermediate levels. In addition, the expression of *MTOR* was significantly higher in D2 and D3 than in D0 and D1 (*p* < 0.05), with no significant difference between D2 and D3 or between D0 and D1 (*p* > 0.05). Overall, these results suggest that moderate DSPM replacement, especially at the D2 level, promoted a more favorable molecular state for purine turnover, methyl donor metabolism, amino acid transport, and anabolic signaling in muscle.

### 3.9. Expression of Muscle Quality Related Genes

**Figure 9:**
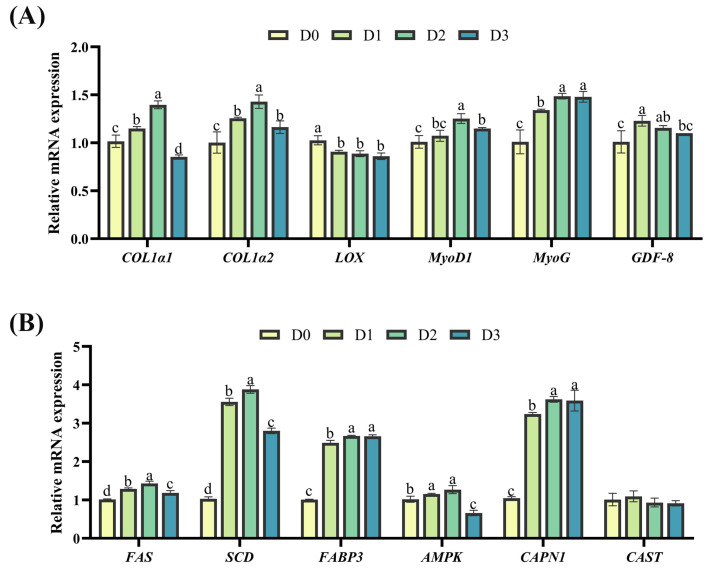
Relative expression levels of genes related to muscle and collagen synthesis, myogenic regulation, lipid metabolism and proteolysis between different groups. (**A**) Relative mRNA expression levels of genes related to collagen synthesis and myogenic regulation. (**B**) Relative mRNA expression levels of genes related to lipid metabolism and proteolysis. Notes: Data were presented as the mean ± SD, n = 3. Superscripts with different lowercase letters indicate significant differences between groups (*p* < 0.05).

As shown in Figure 9A, dietary DSPM replacement significantly affected the expression of genes related to collagen remodeling and myogenic regulation (*p* < 0.05). Overall, the D2 group exhibited the most favorable expression pattern for muscle structural formation, as reflected by the highest expression of *COL1α1*, *COL1α2*, and *MyoD1*. Among the collagen-related genes, *COL1α1* showed the highest level in D2 and the lowest level in D3, whereas *COL1α2* was significantly upregulated in D2, with D1 and D3 showing intermediate levels that remained higher than D0. In contrast, the collagen cross-linking-related gene *LOX* was significantly higher in D0 than in the DSPM replacement groups, indicating an overall downregulation after DSPM inclusion. For myogenic regulatory genes, *MyoD1* was highest in D2, followed by D3, whereas *MyoG* was significantly upregulated in all DSPM replacement groups relative to D0. The myogenesis-inhibitory gene *GDF-8* showed the highest expression in D1, an intermediate level in D2, and the lowest level in D0. Together, these results suggest that moderate DSPM replacement, particularly in D2, was more conducive to coordinated collagen deposition and myogenic regulation in muscle.

As shown in Figure 9B, genes related to lipid metabolism, metabolic regulation, and protein degradation differed significantly among groups (*p* < 0.05). Overall, the D2 group showed the most favorable expression pattern, characterized by enhanced transcription of genes involved in fatty acid synthesis, lipid transport, energy-sensing regulation, and proteolytic remodeling. Specifically, the lipogenic genes *FAS* and *SCD* both reached their highest expression levels in D2, with *SCD* showing a marked decline in D3 relative to D1 and D2. The fatty acid transport-related gene *FABP3* was significantly upregulated in all DSPM replacement groups compared with D0. In addition, the energy metabolism-related gene *AMPK* showed the highest expression in D2, followed by D1 and D0, and the lowest level in D3, indicating that moderate DSPM replacement was more conducive to metabolic regulation, whereas excessive replacement weakened this response. The protein hydrolysis-related gene *CAPN1* was significantly elevated in D2 and D3, whereas no significant difference was detected in the expression of its endogenous inhibitor *CAST* among groups (*p* > 0.05). Together, these results indicate that moderate DSPM replacement promoted a more favorable molecular state for intramuscular lipid metabolism, metabolic homeostasis, and controlled proteolytic remodeling.

**Figure 10:**
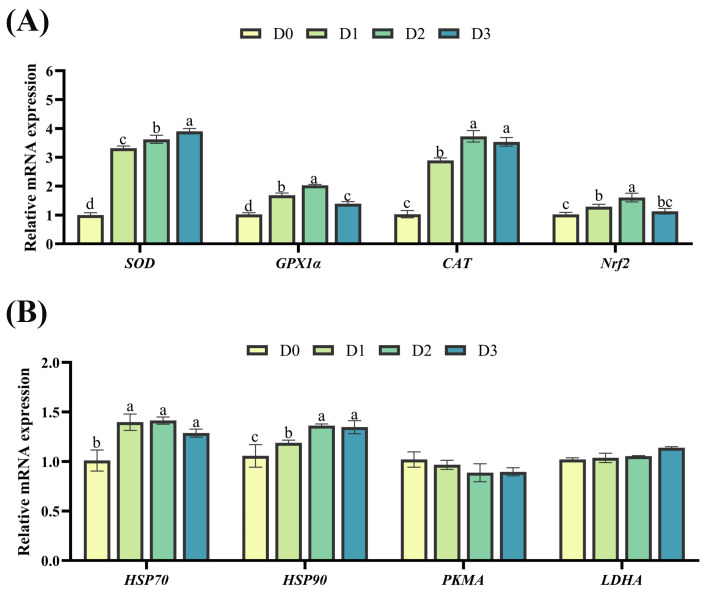
Relative mRNA expression levels of genes related to antioxidant defense, stress response, and glycolysis in muscle of different groups. (**A**) Relative mRNA expression levels of genes related to antioxidant defense. (**B**) Relative mRNA expression levels of genes related to stress response and glycolysis. Notes: Data were presented as the mean ± SD, n = 3. Superscripts with different lowercase letters indicate significant differences between groups (*p* < 0.05).

Significant differences were also observed in the expression of antioxidant and stress-related genes among groups (Figure 10, *p* < 0.05). Overall, DSPM replacement enhanced the antioxidant defense response in muscle, with D2 showing the most balanced expression pattern, whereas D3 displayed a stronger stress-responsive profile. Among the antioxidant-related genes (Figure 10A), *SOD* expression increased progressively with increasing DSPM replacement and reached its highest level in D3. In contrast, *GPX1α* and *Nrf2* both showed their highest expression in D2, while *CAT* was significantly upregulated in D2 and D3 compared with D0 and D1. Among the stress- and glycolysis-related genes (Figure 10B), *HSP70* was significantly higher in all DSPM replacement groups than in D0, with no significant differences among D1, D2, and D3, whereas *HSP90* was significantly increased in D2 and D3 and showed an intermediate level in D1. By contrast, no significant differences were detected in the expression of the glycolytic genes *PKMA* and *LDHA* among groups (*p* > 0.05). Taken together, these results suggest that moderate DSPM replacement strengthened antioxidant regulation without causing marked glycolytic disturbance, whereas excessive replacement induced a more pronounced stress response pattern.

### 3.10. Correlation Analysis

**Figure 11:**
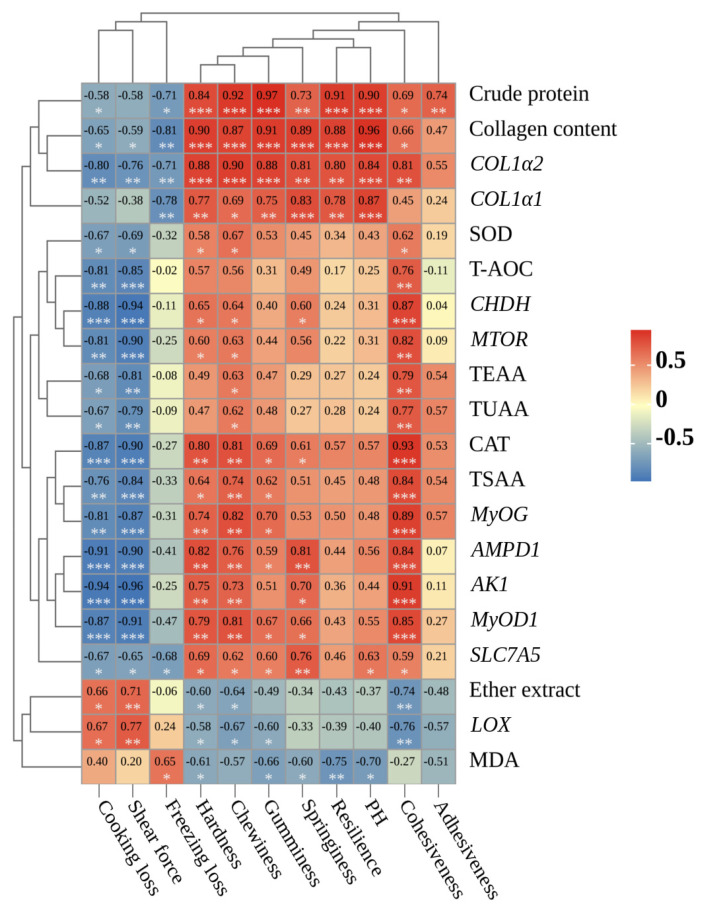
Heatmap of correlations between flesh quality traits and nutritional components and flesh quality-related gene expression. Notes: The abscissa represents flesh quality traits, the ordinate represents nutritional content and genes related to flesh quality, and the color represents the direction of correlation. Asterisks indicate significant correlations (*: *p* < 0.05, **: *p* < 0.01, ***: *p* < 0.001).

As shown in Figure 11, the Pearson correlation heatmap combined with hierarchical clustering revealed systematic associations among muscle quality phenotypes, nutritional composition, antioxidant status, amino acid levels, and the expression of quality-related genes. Overall, cooking loss, shear force, and freezing loss were clustered into a negative-quality module, whereas hardness, chewiness, gumminess, springiness, resilience, cohesiveness, adhesiveness, and pH were clustered into a positive-quality module. These two modules showed clearly opposite correlation patterns. The negative-quality module (cooking loss, shear force, and freezing loss) generally showed negative correlations with nutritional and structural indicators, including crude protein, collagen content, *COL1α2*, and *COL1α1*, antioxidant indices, including SOD, CAT, and T-AOC, amino acid-related parameters, including TEAA, TUAA, and TSAA, and genes associated with improved muscle quality, including *AMPD1*, *AK1*, *CHDH*, *SLC7A5*, *MTOR*, *MyoD1*, and *MyoG*. In contrast, this module tended to show positive correlations with lipid deposition and deterioration-related factors, including ether extract, *LOX*, and MDA. By contrast, the positive-quality module showed an overall positive correlation trend with nutritional and structural indicators (crude protein, collagen content, *COL1α2*, and *COL1α1*), antioxidant indices (SOD, CAT, and T-AOC), amino acid-related parameters (TEAA, TUAA, and TSAA), and muscle quality-improving genes (*AMPD1*, *AK1*, *CHDH*, *SLC7A5*, *MTOR*, *MyoD1*, and *MyoG*), while showing negative correlations with lipid deposition and unfavorable factors (ether extract, *LOX*, and MDA).

## 4. Discussion

With the development of novel animal protein sources, muscle quality has become an important focus in fish nutrition research [20]. FM replacement strategies usually show a clear dose-dependent effect, whereby moderate replacement can maintain or even improve growth performance and flesh quality, whereas excessive replacement reduces nutrient utilization efficiency, aggravates oxidative stress, and impairs tissue structure [21]. When the level of fermented silkworm pupae meal replacing FM exceeded 40%, the growth performance, feed utilization, and condition indices of largemouth bass all declined [22]. Li et al. also reported that dietary supplementation with silkworm pupae meal in mandarin fish (*Siniperca chuatsi*) enhanced intestinal digestion, metabolism, and immunity, and significantly increased the levels of essential fatty acids in muscle, thereby improving flesh quality [7]. However, to date, there have been no reports on the application of DSPM in pearl gentian grouper. Good growth performance is the basis for evaluating flesh quality. In the present study, the D2 group exhibited the best overall growth performance, improving FBW, WGR and SGR while maintaining SR and reducing FCR. In contrast, the complete replacement group (D3) showed reduced SR and elevated FCR. This inferior performance was likely associated with multiple factors rather than chitin alone, including reduced nutrient digestibility, possible amino acid imbalance, lower bioavailability of some nutrients, altered lipid metabolism, and increased metabolic and oxidative stress under complete FM replacement. On the one hand, chitin present in insect protein sources may reduce nutrient digestibility and feed utilization efficiency [23,24]. On the other hand, complete replacement of FM by DSPM may also alter the balance and availability of essential nutrients, increase the metabolic cost of nutrient assimilation and utilization, and aggravate redox imbalance, as indicated by the higher FCR, reduced survival, increased MDA content, impaired muscle structure, and less favorable water-holding capacity observed in D3. Therefore, the poorer growth and flesh quality in D3 likely reflected a systemic nutritional and metabolic limitation under complete FM replacement, rather than the effect of chitin alone.

From the perspective of conventional muscle composition, the beneficial effects of DSPM are reflected not merely in the improvement of a single nutritional index, but more importantly in its promotion of a nutrient deposition pattern that is more favorable for flesh quality [25]. Fish flesh quality is generally determined by myofibrillar proteins and connective tissue collagen, and higher protein deposition together with an appropriate collagen content helps provide better structural support and tissue integrity [26]. Tong et al. found in triploid crucian carp that partial replacement of FM with fish by-product hydrolyzed collagen improved muscle quality and metabolic status [27]. Castro et al. also reported that under high plant-protein diets, hydroxyproline supplementation promoted collagen deposition and improved muscle growth and texture [28]. Therefore, the higher crude protein and collagen contents observed in the D1 and D2 groups may have provided an important structural basis for the improvement in WHC and textural properties, whereas the decline in collagen content in D3 back to the control level may suggest that complete replacement failed to maintain a stable structural basis for muscle tissue.

Regarding changes in muscle amino acid composition, the D2 group was particularly notable for amino acids associated with muscle structure formation and flavor development, such as Pro, Ser, and Thr. Although the D3 group exhibited higher levels of Asp, Glu, and several essential amino acids, its collagen content did not show a corresponding advantage. This indicates that the formation of fish flesh quality depends not only on the absolute accumulation of amino acids, but also on whether these amino acids can be effectively utilized for muscle tissue construction and the optimization of related metabolic processes [29]. Pro and Hyp are important substrates for collagen biosynthesis [30], while Ser and Thr are also involved in cellular metabolic regulation, protein translation, and tissue renewal [31]. Therefore, the higher Pro level in the D2 group may indicate more active collagen synthesis and extracellular matrix remodeling. Free amino acids are key chemical markers for evaluating fish flavor quality. Specifically, Glu and Asp are the major contributors to umami taste, Thr, Gly, Ser, Ala, Lys, and Pro mainly contribute to sweetness, whereas Leu, Val, Ile, His, Phe, Met, and Arg are mainly associated with bitterness [32]. Previous studies have shown that adding DSPM to broiler diets can maintain amino acid contents comparable to those of the control group [33]. In the present study, TAA, TEAA, and TUAA generally increased with increasing DSPM levels, whereas TSAA was higher in the DSPM-fed groups than in D0 but did not show a strictly dose-dependent increase, indicating that replacing FM with DSPM had a certain effect on the flavor characteristics of grouper muscle. Taken together, the advantage of the D2 group lies not only in its higher amino acid levels, but also in its ability to more effectively convert amino acid supply into structural and metabolic advantages, thereby promoting the formation of high-quality muscle with the potential to improve flesh flavor. In contrast, although D3 showed some advantages over D2 in flavor-related amino acids, according to the proximate composition results, it failed to maintain adequate collagen content to build a stable muscle structure.

n-3 PUFAs play important preventive and regulatory roles in reducing the risk of certain diseases by modulating processes related to atherosclerosis and myocardial infarction [34]. The fatty acid profile indicated that DSPM could, to a certain extent, improve the nutritional lipid composition of muscle and enhance its potential health value. Previous studies have shown that the fatty acid composition of fish tissues is largely influenced by the dietary lipid source and composition, while also being regulated by selective deposition, biotransformation, and metabolic processes; therefore, dietary changes usually lead to alterations in the muscle fatty acid profile [35]. Specifically, the D1 group showed the best performance in n-3 PUFA and DHA levels, while the D2 group largely maintained DHA content comparable to that of the D0 group. In contrast, the D3 group showed a declining trend in DHA content and the n-3/n-6 ratio, while n-6 PUFA continued to increase. Considering that EPA and DHA are important n-3 long-chain polyunsaturated fatty acids in fish, involved in development, metabolism, and immune regulation, and are also key indicators for evaluating the nutritional value of fish flesh, these results reveal clear differences in the effects of different DSPM replacement levels on muscle lipid quality [36]. However, improvement in the nutritional characteristics of fatty acids does not necessarily mean a synchronous enhancement in overall muscle quality. Unsaturated fatty acids, especially PUFAs, although nutritionally valuable, are more susceptible to oxidation. Lipid oxidation can further induce protein oxidation, thereby weakening WHC and accelerating flesh quality deterioration [37]. Combined with the decline in growth performance and the proximate composition results observed in the D3 group, it can be inferred that although high replacement levels showed certain nutritional advantages in lipid composition, they may also increase oxidative risk, thereby leading to reduced flesh quality. In addition, both AI and TI, classical indices based on fatty acid composition for assessing the potential atherogenicity and thrombogenicity of foods, were reduced, further indicating that DSPM improved the health-related lipid characteristics of muscle [38]. Therefore, the regulation of muscle fatty acid profiles by DSPM may have dual effects: an appropriate replacement level is more conducive to balancing lipid nutritional value and oxidative stability, whereas an excessively high replacement level may impair flesh quality due to changes in fatty acid composition and aggravated oxidative stress.

Muscle physicochemical properties are important indicators for evaluating the WHC, structural integrity, and textural quality of fish flesh [39]. In general, reductions in cooking loss, freezing loss, and shear force reflect enhanced WHC and structural stability of muscle tissue [40,41]. Microstructural damage during frozen storage aggravates water migration and quality deterioration; therefore, increased freezing loss usually indicates reduced muscle tissue stability [42]. In the present study, the D2 group exhibited markedly better WHC than the other treatment groups. Although the D3 group showed lower cooking loss than the control group, its freezing loss was higher than that of the other DSPM-treated groups, indicating that an appropriate dietary replacement level of DSPM improved muscle WHC, whereas the D3 group failed to maintain an optimized and stable state, possibly due to impaired metabolism and antioxidant capacity. Previous studies have shown that muscle pH is closely related to WHC and texture, and lower pH is usually accompanied by greater water loss, poorer textural properties, and accelerated flesh deterioration [43]. In the present study, D1 and D2 showed significantly higher thawed muscle pH values than the control group, whereas D3 did not differ significantly from D0, suggesting that DSPM may have alleviated the tendency of postmortem flesh deterioration caused by pH decline. In addition, nutritional regulation can improve muscle hardness and chewiness by promoting collagen deposition between muscle fibers [44]. The physicochemical results of this study indicate that moderate DSPM treatment effectively maintained the structural stability of muscle proteins while preserving tenderness, thereby retaining the intrinsic delicate texture of fish flesh. Notably, the improvement was most pronounced in the D2 group, whereas the D3 group showed a declining trend in these indices, which is consistent with our previous inference that complete DSPM replacement impairs flesh quality.

Differences in muscle histology are usually closely associated with the formation of fish flesh texture. Muscle fiber morphology is also a key determinant of textural characteristics. Higher muscle fiber density is generally associated with greater hardness, elasticity, and chewiness, whereas larger fiber diameter is usually unfavorable for texture formation [45]. Li et al. reported that appropriate dietary composition could improve flesh hardness and shear force by regulating muscle fiber morphology, thereby further enhancing muscle quality [46]. Under stress or other adverse conditions, fish usually exhibit muscle fiber damage, loosened tissue structure, texture softening, and reduced WHC, and these changes are usually interrelated rather than independent [47]. Samples with more compact muscle fiber arrangement and narrower inter-fiber spaces are more likely to exhibit stable tissue structure and superior textural performance [48]. In the present study, compared with the D0 group, the D1 and D2 groups showed clearer muscle fiber boundaries, more compact arrangement, and higher muscle fiber density. In contrast, the D3 group displayed irregular fiber cross-sections, loose fiber arrangement, widened myoseptal spaces, reduced muscle fiber density, and increased fiber diameter. Combined with the previous results, these structural differences are closely related to changes in flesh quality. However, whether these differences are mainly driven by muscle fiber growth patterns, connective tissue remodeling, or the combined effects of other metabolic processes still requires further molecular evidence.

The results of muscle antioxidant enzyme activities showed that differences among DSPM-treated groups were not only reflected in antioxidant enzyme activities themselves, but more importantly in whether oxidative damage was effectively controlled. SOD and CAT represent antioxidant defense responses, whereas MDA more directly reflects lipid peroxidation damage. Therefore, only when enhanced antioxidant enzyme activities are accompanied by reduced MDA can the redox status be considered more stable [49]. In *Pangasianodon hypophthalmus*, elevated MDA was associated with impaired muscle quality, indicating that lipid peroxidation itself may be a major factor affecting flesh quality [50]. This may help explain the difference between D2 and D3: in the D2 group, antioxidant defense was effectively activated and successfully suppressed oxidative damage, whereas in the D3 group, the observed antioxidant response was more likely a compensatory reaction to elevated oxidative stress. However, this response appeared insufficient to prevent the accumulation of lipid peroxidation, thereby resulting in reduced flesh quality. This interpretation is consistent with the findings of Fang et al., who reported that antioxidant responses and quality deterioration may occur simultaneously under stress conditions, and that upregulation of antioxidant enzyme activity does not necessarily indicate improved tissue status [51]. Thus, a more stable redox state may help maintain the integrity of muscle fiber structure, whereas the accumulation of oxidative damage may promote tissue loosening accompanied by quality decline.

The contribution of metabolite changes to flesh quality is likely reflected in the coordinated regulation of multiple metabolic modules. Based on the metabolomic results, the improvement in muscle quality of pearl gentian grouper by DSPM appears to be mainly associated with enhanced osmotic homeostasis, optimized collagen/connective tissue metabolism, increased accumulation of umami-related nucleotides, and improved membrane lipid and redox balance. In the D2 group, changes in betaine and related metabolites suggest enhanced osmotic regulation, which may help improve WHC. As an important osmoprotectant and methyl donor, betaine contributes to cellular water balance, protein stabilization, and antioxidant regulation, and is therefore closely associated with reduced water loss and improved flesh quality [52,53]. Taurine likely played a similar role through its functions in osmotic regulation, membrane stabilization, ion balance, calcium homeostasis, and antioxidant defense [54,55]. Together, these metabolic changes may have contributed to improved water retention, tenderness, and juiciness in D2. Changes in hydroxyproline, D-proline, and related metabolites further suggest that DSPM regulated collagen and extracellular matrix metabolism, which may be an important basis for texture improvement. Because collagen and its spatial organization strongly influence muscle hardness, elasticity, thermal stability and palatability [56], the increased collagen content but reduced shear force observed in D2 suggests that DSPM promoted more orderly collagen remodeling rather than excessive deposition of rigid connective tissue. Such remodeling may help maintain tissue support while reducing water loss during processing, thereby improving eating quality. This interpretation is consistent with previous studies showing that fish flesh texture depends not only on total collagen content, but also on collagen crosslinking, deposition pattern, and muscle fiber organization [57]. From the perspective of flavor quality, the increase in IMP and related purine nucleotide metabolites in D0 vs. D2 suggests that DSPM also enhanced flavor potential by promoting the accumulation of umami precursors. Because IMP is a key umami nucleotide in fish and can act synergistically with compounds such as glutamate [58], its accumulation indicates that the beneficial effect of DSPM was not limited to texture and WHC, but also extended to flavor-related biochemical potential. KEGG enrichment further showed that D0 vs. D2 was mainly associated with glycerophospholipid metabolism, the pentose phosphate pathway, and carbon metabolism, suggesting coordinated regulation of membrane lipid homeostasis and redox balance. As major structural components of cell membranes, glycerophospholipids reflect membrane integrity, lipid turnover, and lipid oxidation status, all of which are closely related to water retention, texture stability, and flavor development [59]. Meanwhile, the pentose phosphate pathway provides NADPH for antioxidant defense, thereby helping maintain the redox homeostasis of cell membranes and myofibrillar proteins [60]. These results suggest that the superior flesh quality of D2 was associated with a more favorable metabolic state for membrane protection and antioxidant support. In contrast, metabolites such as methionine sulfoxide and 3-methylhistidine in D3 suggest more pronounced oxidative modification and protein turnover at the high replacement level. Because methionine sulfoxide is an indicator of oxidative protein damage and 3-methylhistidine is associated with myofibrillar protein degradation [61,62,63], these changes imply that excessive DSPM inclusion imposed additional oxidative and proteolytic stress, thereby weakening its beneficial effects on flesh quality. Overall, the effect of DSPM on muscle quality showed a clear dose-dependent pattern: an appropriate replacement level improved tissue stability, water-holding capacity, and flavor potential, whereas excessive replacement induced metabolic stress responses unfavorable to further quality improvement. Thus, the superior muscle quality in D2 was most likely associated with coordinated optimization of osmotic homeostasis, collagen remodeling, flavor precursor accumulation, and membrane lipid/redox balance, whereas D3 failed to maintain the stability of these quality-related metabolic processes.

Combined with muscle quality phenotypes and gene expression results, the improvement in muscle quality induced by DSPM in pearl gentian grouper appears to be mainly associated with the coordinated regulation of energy metabolism, nutrient sensing, muscle fiber/collagen remodeling, and stress defense. The differential expression of *AMPD1*, *AK1*, and *NT5E* suggests that DSPM reshaped purine nucleotide turnover, which may contribute to both muscle energy homeostasis and the accumulation of umami-related precursors such as IMP [64,65]. The upregulation of *SLC7A5* and *MTOR* further indicates enhanced amino acid transport and anabolic capacity, providing a molecular basis for protein deposition and tissue integrity [66,67]. Increased *CHDH* expression suggests enhanced conversion of choline to betaine, which may improve myocyte hydration and membrane stability, thereby supporting WHC [68]. This interpretation is also consistent with the lack of significant changes in *PKMA* and *LDHA* in the D2 group despite its superior pH and water-holding performance [69]. In addition, the expression patterns of *COL1α1*, *COL1α2*, *MyoD1*, and *MyoG* suggest that DSPM promoted both muscle fiber formation and extracellular matrix remodeling [70,71]. Because the contribution of collagen to flesh quality depends not only on its amount but also on its deposition pattern and crosslinking status [72], the upregulation of collagen-related genes together with reduced shear force in D2 indicates improved tissue support without excessive hardening. Consistently, the downregulation of *LOX*, a key enzyme involved in collagen crosslinking, may have helped avoid excessive tissue stiffening [73,74]. Meanwhile, the moderate expression of *GDF-8* in D2 suggests that DSPM maintained a balance between myogenic promotion and inhibition. The responses of *FAS*, *SCD*, and *FABP3* further imply improved intramuscular lipid metabolism, which may contribute to better juiciness, tenderness, and flavor release [75,76]. Likewise, the upregulation of *CAPN1* with relatively stable *CAST* expression suggests a more favorable proteolytic potential for limited myofibrillar degradation and texture softening [77,78]. Finally, antioxidant- and stress-related genes indicate that the beneficial effect of DSPM on flesh quality also depended on maintenance of redox homeostasis [79]. The upregulation of *Nrf2* and its downstream targets *SOD*, *CAT*, and *GPX1α*, together with increased expression of *HSP70* and *HSP90*, suggests enhanced antioxidant defense and protein homeostasis in muscle. Such coordinated protection may help preserve membrane integrity, reduce oxidative damage to lipids and proteins, and maintain WHC and tenderness. Although stress-protective signals such as *SOD* and *CAT* also remained elevated in D3, its flesh quality was not superior to that of D2, implying that the response in D3 may reflect stress adaptation rather than an optimal quality state. Overall, the superior flesh quality of the D2 group was likely associated with the coordinated optimization of purine metabolism, *MTOR*/*AMPK* related energy sensing, muscle fiber and collagen remodeling, and *Nrf2*–*HSP* mediated antioxidant protection.

The correlation heatmap revealed coordinated variation among biological processes involved in muscle quality formation. Crude protein, collagen content, and *COL1α1*/*COL1α2* were positively associated with the positive-quality module and negatively associated with water loss and shear force, suggesting that DSPM-induced flesh quality improvement was supported by enhanced myofibrillar protein deposition and extracellular matrix remodeling. More organized collagen deposition likely helped maintain tissue integrity and inter-fiber structure, thereby improving WHC and textural properties. This is consistent with previous studies showing that fish flesh quality depends not only on collagen content, but also on its distribution, maturation, and interaction with the myofibrillar network [80]. Accordingly, the closer association of *LOX* with the negative-quality module suggests that quality differences depend less on collagen quantity alone than on whether collagen remodeling produces a stable but not excessively rigid structure. SOD, CAT, and T-AOC clustered with the positive-quality module, whereas MDA showed the same directional pattern as water loss and shear force, indicating that redox homeostasis is important for maintaining these structural advantages. Enhanced antioxidant capacity may preserve membrane integrity, water distribution, and tissue stability by limiting oxidative damage to membrane lipids and myofibrillar proteins, whereas MDA accumulation reflects aggravated lipid peroxidation and is associated with structural damage and texture deterioration [81]. In addition, the clustering of TEAA, TUAA, TSAA with *SLC7A5*, *MTOR*, *MyoD1*, *MyoG*, *AMPD1*, *AK1*, and *CHDH* suggests close coupling among amino acid supply, nutrient sensing, myogenic regulation, and energy metabolism. This implies that amino acids were not only elevated, but also more effectively utilized for protein synthesis, muscle fiber formation, tissue renewal, and osmotic homeostasis. Therefore, superior muscle quality appears to result from the coordinated integration of structural support, oxidative balance, and synchronized nutrient sensing and anabolism, which may explain why the D2 group showed lower water loss, lower shear force, and better textural properties.

It should also be noted that the present study has some limitations. Although flavor-related amino acids and IMP-related metabolites were analyzed, sensory evaluation and volatile-compound profiling were not performed. Therefore, the observed changes should be interpreted as improvements in flavor-related biochemical potential rather than direct evidence of enhanced perceived taste or odor. In addition, instrumental muscle color parameters were not assessed in this experiment, although color is an important consumer-relevant quality attribute. Future studies should include sensory evaluation, volatile aroma compound analysis, taste-active compound profiling, and muscle color measurement to determine whether DSPM-induced biochemical and structural changes can be translated into perceptible improvements in taste, odor, appearance, and overall consumer acceptance.

In conclusion, moderate DSPM inclusion improved the flesh quality of pearl gentian grouper, with 50% FM replacement producing the most favorable effects on texture, WHC, structural stability, and flavor-related biochemical potential. These improvements were associated with coordinated changes in muscle structure, antioxidant status, and metabolic regulation related to osmotic balance, collagen remodeling, and purine metabolism. In contrast, complete FM replacement impaired flesh quality. Therefore, DSPM can be considered a promising alternative protein source for improving muscle quality in pearl gentian grouper when used at an appropriate inclusion level.

## 5. Conclusions

Dietary DSPM improved the flesh quality of pearl gentian grouper in a replacement-level-dependent manner, with 50% fishmeal replacement showing the best overall performance. This level enhanced muscle protein and collagen deposition, water-holding capacity, texture, muscle fiber organization, antioxidant stability, flavor-related amino acid accumulation, and lipid nutritional value. These benefits were associated with coordinated regulation of collagen remodeling, osmotic balance, purine metabolism, lipid homeostasis, myogenesis, and antioxidant defense. In contrast, complete fishmeal replacement weakened muscle structure and redox balance. Therefore, 50% replacement of fishmeal with DSPM is recommended as an effective strategy to improve grouper flesh quality under the present conditions.

## Figures and Tables

**Table 1:** Growth performance of pearl gentian grouper fed different levels of DSPM diets. Values are expressed as mean ± standard deviation (SD), “±” means plus or minus.

Parameters	D0	D1	D2	D3
IBW (g)	12.85 ± 0.01	12.84 ± 0.01	12.84 ± 0.00	12.84 ± 0.01
FBW (g)	67.71 ± 0.63 ^c^	72.88 ± 0.89 ^b^	79.68 ± 1.13 ^a^	67.61 ± 1.14 ^c^
WGR (%)	427.06 ± 5.15 ^c^	467.43 ± 6.96 ^b^	520.54 ± 8.83 ^a^	426.58 ± 8.57 ^c^
SGR (% day^−1^)	2.97 ± 0.02 ^c^	3.10 ± 0.02 ^b^	3.26 ± 0.03 ^a^	2.97 ± 0.03 ^c^
FCR	1.05 ± 0.02 ^b^	1.00 ± 0.02 ^c^	0.98 ± 0.01 ^c^	1.34 ± 0.03 ^a^
SR (%)	100.00 ± 0.00 ^a^	100.00 ± 0.00 ^a^	100.00 ± 0.00 ^a^	79.00 ± 1.73 ^b^

Notes: IBW: Initial body weight; FBW: Final weight; WGR: Weight gain rate; SGR: Specific growth rate; FCR: Feed conversion ratio; SR: Survival rate. Data are expressed as Mean ± SD, n = 3. Different lowercase letters in superscript indicate significant differences between groups (*p* < 0.05).

**Table 2:** The proximate composition of pearl gentian grouper. Values are expressed as mean ± SD, “±” means plus or minus.

Parameters	D0	D1	D2	D3
Moisture (%)	75.32 ± 1.32 ^b^	76.30 ± 0.39 ^ab^	76.98 ± 0.04 ^a^	77.13 ± 0.10 ^a^
Crude protein (%)	86.86 ± 0.11 ^c^	87.81 ± 0.09 ^a^	87.88 ± 0.02 ^a^	87.20 ± 0.13 ^b^
Ether extract (%)	5.45 ± 0.08 ^a^	5.34 ± 0.07 ^ab^	5.29 ± 0.06 ^b^	5.28 ± 0.04 ^b^
Ash (%)	6.55 ± 0.01 ^b^	6.53 ± 0.03 ^b^	6.59 ± 0.01 ^a^	6.60 ± 0.01 ^a^
Collagen content (μg/mg)	5.95 ± 0.17 ^c^	8.48 ± 0.20 ^b^	10.58 ± 0.23 ^a^	5.98 ± 0.18 ^c^

Notes: Crude protein, ether extract and ash are calculated based on dry matter. Data are expressed as Mean ± SD, n = 3. Different lowercase letters in superscript indicate significant differences between groups (*p* < 0.05).

**Table 3:** Physicochemical characteristics of pearl gentian grouper. Values are expressed as mean ± SD; “±” means plus or minus.

Parameters	D0	D1	D2	D3
Cooking loss (%)	25.56 ± 1.07 ^a^	24.03 ± 0.65 ^b^	19.92 ± 0.70 ^d^	22.22 ± 0.47 ^c^
Freezing loss (%)	5.72 ± 0.30 ^ab^	5.21 ± 0.23 ^bc^	5.04 ± 0.06 ^c^	5.88 ± 0.41 ^a^
Shear force (N)	4.24 ± 0.08 ^a^	3.84 ± 0.07 ^b^	3.06 ± 0.06 ^d^	3.36 ± 0.09 ^c^
pH after thawing	5.99 ± 0.04 ^c^	6.25 ± 0.02 ^b^	6.35 ± 0.04 ^a^	6.02 ± 0.06 ^c^

Notes: Data are expressed as Mean ± SD, n = 3. Different lowercase letters in superscript indicate significant differences between groups (*p* < 0.05).

**Table 4:** Quantification of muscle tissue slice parameters. Values are expressed as mean ± SD; “±” means plus or minus.

Parameters	D0	D1	D2	D3
Muscle fiber number	113.00 ± 2.65	117.33 ± 1.53	114.67 ± 2.52	113.00 ± 2.00
Muscle fiber density (n/mm^2^)	426.94 ± 6.34 ^bc^	454.21 ± 9.69 ^a^	438.30 ± 6.92 ^b^	415.53 ± 9.90 ^c^
Muscle fiber diameter (μm)	70.63 ± 1.03 ^b^	64.89 ± 1.60 ^c^	69.45 ± 2.23 ^b^	75.43 ± 3.68 ^a^

Notes: Data are expressed as Mean ± SD, n = 3. Different lowercase letters in superscript indicate significant differences between groups (*p* < 0.05).

**Table 5:** Differential metabolite annotation table.

**D0 vs. D2**			
**MS2 Name**	**Class**	**log_2_ FC**	**VIP**
N, N-Diethyl-2-aminoethanol	Organonitrogen compounds	−9.73	19.35
D-proline	Carboxylic acids and derivatives	1.18	15.94
Inosine 5′-monophosphate	Purine nucleotides	1.57	8.39
Pregabalin	Carboxylic acids and derivatives	2.44	8.13
Inosine 5′-phosphate	Purine nucleotides	1.39	8.01
5-[(4-Nitrophenoxy) methyl]-1,3-oxazolidin-2-one	Benzene and substituted derivatives	3.12	6.62
D-mannose 1-phosphate	Organooxygen compounds	1.34	6.51
**D0 vs. D3**			
**MS2 Name**	**Class**	**log_2_ FC**	**VIP**
Pregabalin	Carboxylic acids and derivatives	2.88	10.12
5-[(4-Nitrophenoxy) methyl]-1,3-oxazolidin-2-one	Benzene and substituted derivatives	3.64	8.79
Ethyl sulfate	Organic sulfuric acids and derivatives	−1.01	5.94
Methionine sulfoxide	Carboxylic acids and derivatives	3.10	5.60
2-Hydroxy-6-methylpyridine-4-carboxylic acid	Pyridines and derivatives	−2.98	5.57
D-erythro-Imidazolylglycerol phosphate	Organic phosphoric acids and derivatives	−2.00	5.44
Flonicamid	Pyridines and derivatives	−2.40	5.08
3-methylhistidine	Carboxylic acids and derivatives	−1.56	5.08
**D2 vs. D3**			
**MS2 Name**	**Class**	**log_2_ FC**	**VIP**
D-erythro-Imidazolylglycerol phosphate	Organic phosphoric acids and derivatives	−2.41	7.38
D-mannose 1-phosphate	Organooxygen compounds	−1.36	6.82
Acetyl-beta-alanine	Carboxylic acids and derivatives	−1.35	5.96
2-(4-Phenylphenyl)-N-(pyridin-2-yl) acetamide	Benzene and substituted derivatives	−1.03	5.10
8S-Hydroxy-5Z,9E,11Z,14Z-eicosatetraenoic acid	Fatty Acyls	−1.39	5.04
Cinchonine	Cinchona alkaloids	−1.05	5.02

## Data Availability

The data presented in this study are available on request from the corresponding authors due to privacy and confidentiality restrictions.

## Supplementary Materials

The following supporting information can be downloaded at: https://www.mdpi.com/article/10.3390/foods15152640/s1, Table S1. Formulation and nutrient composition of the experimental diets (%, dry matter); Table S2. qPCR primers used in this study; Table S3. Muscle amino acid composition of groupers fed different diets (%, dry matter; Values are expressed as mean ± SD, “±” means plus or minus); Table S4. Muscle fatty acid composition of groupers fed different diets (%, dry matter; Values are expressed as mean ± SD, “±” means plus or minus); Table S5. Statistical analysis of pearl gentian grouper metabolome assembly.
