# Supplementary material for: Muscle Quality Responses of Pearl Gentian Grouper (Epinephelus fuscoguttatus ♀ × Epinephelus lanceolatus ♂) to Fishmeal Replacement by Defatted Silkworm Pupae Meal: Insights from Texture, Histology, Antioxidant Status and Metabolomics
Source: Foods. 2026 Jul 28;15(15):2640. doi: 10.3390/foods15152640 (PMC13465419; doi:10.3390/foods15152640)
Supplement: Supplementary file 1 [file foods-15-02640-s001.zip › foods-4410862-supplementary.pdf]

Table S1 Formulation and nutrient composition of the experimental diets (%, dry matter)

Ingredients	Groups			
	D0	D1	D2	D3
DSPM	0.00	10.66	21.33	42.65
fishmeal	45.00	33.75	22.50	0.00
Soybean meal	10.00	10.00	10.00	10.00
Soy protein concentrate	6.00	6.00	6.00	6.00
Wheat gluten flour	10.00	10.00	10.00	10.00
Flour	16.00	16.00	16.00	16.00
Soya Lecithin	2.00	2.00	2.00	2.00
Soybean oil	2.00	2.30	2.59	3.19
Fish oil	2.00	2.00	2.00	2.00
Multivitamin and Multimineral[1]	1.00	1.00	1.00	1.00
Calcium dihydrogen phosphate	1.80	1.80	1.80	1.80
DMPT	0.10	0.10	0.10	0.10
Antioxidant	0.10	0.10	0.10	0.10
Choline Chloride	0.50	0.50	0.50	0.50
Microcrystalline cellulose	3.50	3.79	4.08	4.66
Total	100.00	100.00	100.00	100.00
Crude protein[2]	48.49	48.49	48.49	48.49
Ether extract[2]	9.64	9.64	9.64	9.64

Notes:[1] The vitamin–mineral premix supplied the following concentrations per kilogram of diet: vitamin A (retinyl acetate), ≥ 675,000 IU; vitamin D3, ≥ 180,000 IU; dl-α-tocopheryl acetate, ≥ 6.0 g; menadione, ≥ 1.2 g; thiamine nitrate, ≥ 0.90 g; riboflavin, ≥ 1.35 g; pyridoxine hydrochloride, ≥ 1.05 g; cyanocobalamin, ≥ 0.0075 g; calcium D-pantothenate, ≥ 4.5 g; nicotinamide, ≥ 6.75 g; folic acid, ≥ 0.375 g; D-biotin, ≥ 0.015 g; L-ascorbyl-2-phosphate (expressed as L-ascorbic acid), ≥ 15.0 g; inositol, ≥ 10.0 g; Fe, 20.0 g; Zn, 9.6 g; Mn, 6.4 g; Cu, 0.60 g; Co, 0.08 g; Se, 0.04 g; I, 0.08 g.

[2]Crude protein and ether extract contents were calculated from ingredient composition values and converted accordingly.

Table S2 qPCR primers used in this study

Gene names	Primers	Sequences (5'-3')	Accession number
β-actin	*β-actin-F*	GGCTACTCCTTCACCACCACA	AY510710.2
	β-actin-R	TCTGGGCAACGGAACCTCT	
AMPD1	*AMPD1-F*	TGCAACCCTAACGAGTGGAC	XM_049581031.1
	AMPD1-R	AAGTGGGGCACAAAGTTCCT	
NT5E	*NT5E-F*	GCTCCACGCTGTTTAAAGCC	XM_049590398.1
	NT5E-R	CTGGCCGTATCGTCTCACTG	
AK1	*AK1-F*	AAAGGCATAGCAAGGGGGAG	XM_049604575.1
	AK1-R	CTCTCTTTTTCGCGCTGTGG	
CHDH	*CHDH-F*	ACCTGGGGCTAGTTTGACCA	XM_049580162.1
	CHDH-R	AGTCACAATTCGTCGGGCTC	
SLC7A5	*SLC7A5-F*	GGATGCAGCGAGGGGTTAAT	XM_049574642.1
	SLC7A5-R	TGCGATGGCTGGAGTGTTAG	
MTOR	*MTOR-F*	GTGACGCCAGTATCCGAGAG	XM_033630095.2
	MTOR-R	GCATGGGGTTGTTTGGTGTC	
COL1α1	*COL1α1-F*	TGACAGCGGAACGGTGATG	XM_033645844.1
	COL1α1-R	CAACCTGAGGCTCCGTGAA	
COL1α2	*COL1α2-F*	AGAGGCTGGACACAGAGGA	XM_049601416.1
	COL1α2-R	ACCGCTCTCACCCTTATCAC	
LOX	*LOX-F*	ACAGAAGGGGAGACATTTGGTC	XM_033618596.2
	LOX-R	TCATCATCTCGACCTCTGACCT	
MyoD1	*MyoD1-F*	AAAGTGTGGAGGCTCGTTCC	XM_033635008.2
	MyoD1-R	GATGAACACTGTGCGAAGCG	
MyoG	*MyoG-F*	GACTGCACTACCGACCCAAC	XM_033625713.2
	MyoG-R	TCACTTGAAGACGACACTCTGG	
GDF-8	*GDF-8-F*	TACGACGTGCTGGGAGATGA	XM_033614507.2
	GDF-8-R	CGACGGACTCGGGTTCAGTG	
FAS	*FAS-F*	ACCTGGACGACACAGTAATTC	XM_033612145.2
	FAS-R	TGTCACCATGTCCACACCATT	
SCD	*SCD-F*	GTTTACAGGCCGAAGCGTTG	XM_033611939.2
	SCD-R	ACATCTGCACCAGGCGTTAT	
FABP3	*FABP3-F*	CGAGAGGGCACAGTAAACCC	XM_033636869.2
	FABP3-R	CCTGGGTTGAGGTGGTGTTA	
AMPK	*AMPK-F*	TAACGGAGAAGATGGCAGAGC	XM_033622315.2
	AMPK-R	CCACTTTATGGCCCGTCAAC	
CAPN1	*CAPN1-F*	GTCTGAAAGCCCAGTCCCTC	XM_033610263.2
	CAPN1-R	TGCGGAACAGGGGAAGTAAC	

Continued from the table above:

CAST	*CAST-F*	AAAAACGATGAAGAGGAGGGAGACA	XM_033627433.2
	CAST-R	TCCTGATGATTCCTTCTCGTACTGT	
SOD	*SOD-F*	CAACCCCCACAACAAGCATC	XM_033633905.2
	SOD-R	TGGCAACATTATCGCCTCCT	
GPX1a	*GPX1a-F*	AGTTCCTGGTCGGTCCTGAT	XM_033625833.2
	GPX1a-R	TGCCACCCAGAGCTTGTTTT	
CAT	*CAT-F*	TTCCTGTCAACTGCCCCTTC	XM_033635388.2
	CAT-R	CCATCACGCTGGTAGTTGGT	
Nrf2	*Nrf2-F*	TGGTGTGGGTGCATTACAGG	XM_033628634.2
	Nrf2-R	CCGGCATAAATGGGATGGCT	
HSP70	*HSP70-F*	GGGGAGCGCAATGTACTCAT	XM_033629352.2
	HSP70-R	TGGTTCACCATCCGGTTGTC	
HSP90	*HSP90-F*	AGCTGATTTCCAACGCCTCA	XM_033634952.2
	HSP90-R	GGTCAGAGACAACAAGCGGA	
PKMA	*PKMA-F*	ATCGGTCCAAGCAGTTCACC	XM_078161273.1
	PKMA-R	AGCATTGAGCTGCTGTGTCT	
LDHA	*LDHA-F*	TAACGTCTACCGAGGAGGCT	XM_033634386.2
	LDHA-R	GAAACGCGAGCTGGAATGTG	

Notes: *β-actin* was used as the reference gene. *β-actin*：*beta-actin*，*AMPD1*：*adenosine monophosphate deaminase 1*，*NT5E*：*5'-nucleotidase ecto*，*AK1*：*adenylate kinase 1*，*CHDH*：*choline dehydrogenase*，*SLC7A5*：*solute carrier family 7 member 5*，*MTOR*：*mechanistic target of rapamycin kinase*，*COL1α1*：*collagen type I alpha 1 chain*，*COL1α2*：*collagen type I alpha 2 chain*，*LOX*：*lysyl oxidase*，*MyoD1*：*myogenic differentiation 1*，*MyoG*：*myogenin*，*GDF-8*：*growth differentiation factor 8*，*FAS*：*fatty acid synthase*，*SCD*：*stearoyl-CoA desaturase*，*FABP3*：*fatty acid binding protein 3*，*CAPN1*：*calpain 1*，*CAST*：*calpastatin*，*SOD*：*superoxide dismutase*，*GPX1α*：*glutathione peroxidase 1 alpha*，*CAT*：*catalase*，*Nrf2*：*NFE2 like bZIP transcription factor 2*，*HSP70*：*heat shock protein 70*，*HSP90*：*heat shock protein 90*，*PKMA*：*pyruvate kinase M1/2a*，*LDHA*：*lactate dehydrogenase. A*

Table S3 Muscle amino acid composition of groupers fed different diets (%, dry matter; Values are expressed as mean ± SD, "±" means plus or minus)

Items	Groups			
	D0	D1	D2	D3
Ile▲■	3.63±0.00^{c}	3.70±0.06^{b}	3.73±0.01^{b}	3.91±0.01^{a}
Leu▲■	6.53±0.02^{c}	6.82±0.11^{b}	6.87±0.03^{b}	7.04±0.02^{a}
Lys▲■	7.69±0.02^{c}	8.04±0.10^{b}	8.08±0.03^{b}	8.28±0.00^{a}
Met▲■	2.64±0.06^{b}	2.75±0.03^{a}	2.81±0.01^{a}	2.82±0.05^{a}
Thr▲♦	3.75±0.00^{b}	3.94±0.10^{a}	3.96±0.04^{a}	3.95±0.00^{a}
Val▲■	3.56±0.02^{c}	3.65±0.04^{b}	3.69±0.01^{b}	3.86±0.04^{a}
His▲■	1.77±0.01^{c}	1.84±0.00^{b}	1.87±0.01^{a}	1.88±0.01^{a}
Phe▲■	3.52±0.03^{c}	3.69±0.07^{b}	3.69±0.05^{b}	3.78±0.03^{a}
Tyr■	3.23±0.02^{b}	3.39±0.10^{a}	3.40±0.01^{a}	3.44±0.05^{a}
Arg▲■	5.15±0.06^{c}	5.33±0.11^{b}	5.45±0.07^{ab}	5.48±0.06^{a}
Asp●	8.60±0.02^{c}	9.01±0.18^{b}	9.04±0.01^{b}	9.28±0.06^{a}
Glu●	12.88±0.03^{c}	13.42±0.22^{b}	13.55±0.04^{b}	13.83±0.04^{a}
Gly♦	4.27±0.13	4.34±0.20	4.43±0.10	4.49±0.12
Ala♦	5.12±0.06^{c}	5.33±0.12^{b}	5.41±0.05^{ab}	5.52±0.08^{a}
Ser♦	3.27±0.01^{b}	3.48±0.10^{a}	3.51±0.03^{a}	3.37±0.04^{b}
Pro♦	2.72±0.09^{c}	2.85±0.14^{bc}	3.15±0.04^{a}	3.02±0.08^{ab}
TAA	78.34±0.51^{c}	81.58±1.68^{b}	82.65±0.26^{ab}	83.95±0.38^{a}
TEAA	38.24±0.19^{c}	39.76±0.62^{b}	40.16±0.02^{b}	41.00±0.09^{a}
TNEAA	40.09±0.32^{c}	41.82±1.06^{b}	42.49±0.28^{ab}	42.96±0.28^{a}
TUAA	21.48±0.05^{c}	22.43±0.40^{b}	22.59±0.05^{b}	23.11±0.10^{a}
TSAA	19.13±0.29^{b}	19.95±0.66^{a}	20.46±0.25^{a}	20.36±0.24^{a}
TBAA	37.73±0.17^{c}	39.20±0.62^{b}	39.60±0.05^{b}	40.48±0.04^{a}

▲: Essential amino acids, EAA; ●: Umami amino acids, UAA; ♦: Sweet amino acids, SAA; ■: Bitter amino acids, BAA. Note: Data are expressed as Mean ± SD, n = 3. Different lowercase letters in superscript indicate significant differences between groups (P<0.05), "±" means plus or minus.

Table S4 Muscle fatty acid composition of groupers fed different diets (%, dry matter; Values are expressed as mean ± SD, "±" means plus or minus)

Items	Groups			
	D0	D1	D2	D3
C14:0	2.25±0.04^{a}	1.60±0.03^{b}	1.47±0.02^{c}	0.82±0.00^{d}
C15:0	0.31±0.01^{a}	0.29±0.01^{b}	0.25±0.00^{c}	0.29±0.00^{b}
C16:0	19.33±0.20^{a}	18.02±0.13^{b}	17.49±0.06^{c}	15.99±0.04^{d}
C17:0	0.52±0.03^{a}	0.41±0.00^{c}	0.47±0.02^{b}	0.36±0.01^{d}
C18:0	8.15±0.00^{d}	8.34±0.01^{c}	8.56±0.03^{b}	9.17±0.01^{a}
C20:0	0.34±0.00^{b}	0.36±0.00^{a}	0.32±0.00^{c}	0.36±0.01^{a}
C24:0	2.68±0.00^{a}	2.41±0.02^{b}	2.21±0.03^{c}	1.76±0.02^{d}
Σ SFA	33.59±0.26^{a}	31.43±0.15^{b}	30.76±0.07^{c}	28.76±0.08^{d}
C16:1n-7	2.28±0.03^{a}	1.66±0.01^{b}	1.58±0.01^{c}	0.98±0.00^{d}
C17:1n-7	0.43±0.01^{a}	0.38±0.02^{b}	0.30±0.01^{c}	0.26±0.01^{d}
C18:1n-9	14.55±0.13^{c}	14.21±0.00^{a}	15.37±0.05^{b}	15.81±0.01^{a}
C20:1n-9	0.78±0.01^{a}	0.70±0.02^{b}	0.78±0.00^{a}	0.64±0.01^{c}
C22:1n-9	1.14±0.00^{a}	1.13±0.04^{a}	0.98±0.04^{b}	1.03±0.03^{b}
Σ MUFA	19.18±0.11^{a}	18.10±0.04^{d}	19.00±0.01^{b}	18.72±0.02^{c}
C18:2n-6	23.74±0.02^{d}	25.26±0.01^{c}	26.41±0.08^{b}	30.89±0.03^{a}
C20:2n-6	0.70±0.01^{b}	0.69±0.02^{b}	0.58±0.01^{c}	0.80±0.00^{a}
C20:4n-6	1.21±0.01^{c}	1.46±0.01^{a}	1.31±0.03^{b}	1.21±0.03^{c}
Σ n-6 PUFA	25.65±0.01^{d}	27.41±0.00^{c}	28.30±0.06^{b}	32.90±0.07^{a}
C18:3n-3	2.27±0.00^{d}	2.68±0.01^{c}	3.33±0.01^{b}	3.76±0.00^{a}
C20:5n-3(EPA)	5.97±0.02^{a}	4.89±0.01^{b}	4.13±0.02^{c}	2.49±0.05^{d}
C22:6n-3(DHA)	14.36±0.14^{b}	15.50±0.10^{a}	14.48±0.01^{b}	13.36±0.04^{c}
Σ n-3 PUFA	22.60±0.16^{b}	23.06±0.12^{a}	21.94±0.02^{c}	19.62±0.00^{d}
n-3/n-6 PUFA	0.88±0.01^{a}	0.84±0.00^{b}	0.78±0.00^{c}	0.60±0.00^{d}
Σ UFA	67.42±0.26^{d}	68.57±0.15^{c}	69.24±0.07^{b}	71.24±0.08^{a}
Σ PUFA	48.24±0.16^{d}	50.48±0.11^{b}	50.24±0.08^{c}	52.52±0.06^{a}
AI	0.42±0.01^{a}	0.36±0.00^{b}	0.34±0.00^{c}	0.27±0.00^{d}
TI	0.33±0.00^{a}	0.30±0.00^{b}	0.31±0.00^{b}	0.30±0.00^{b}

SFA: saturated fatty acids; UFA: unsaturated fatty acid; MUFA: monounsaturated fatty acids; PUFA: polyunsaturated fatty acids. AI: Atherogenicity index; TI: Thrombogenicity index. Note: Data are expressed as Mean ± SD, n = 3. Different lowercase letters in superscript indicate significant differences between groups (P<0.05), "±" means plus or minus.

Table S5 Statistical analysis of pearl gentian grouper metabolome assembly

Type	All	Known	Unknown
POS	5060	894	4166
NEG	6549	940	5609
